# Ultrasound-enhanced gene transfection: vectors, methods, and biomedical applications^☆^

**DOI:** 10.1016/j.ultsonch.2025.107607

**Published:** 2025-10-10

**Authors:** Jie Wu, Yang Gao, Hongju Zhou, Zhengbao Yang, Raul D. Rodriguez, Xiaohui Xu, Lang Ma, Li Qiu

**Affiliations:** a Department of Medical Ultrasound, Frontiers Science Center for Disease-Related Molecular Network, West China Hospital, Sichuan University, Chengdu 610041, China; b Department of Mechanical and Aerospace Engineering, Hong Kong University of Science and Technology, Clear Water Bay, Hong Kong 999077, China; c Research School of Chemistry & Applied Biomedical Sciences, Tomsk Polytechnic University, 30 Lenin Avenue, Tomsk 634050, Russia; d School of New Energy and Materials, Southwest Petroleum University, Chengdu 610500, China

**Keywords:** Ultrasound, Gene therapy, Microbubbles, Sonoporation, Sonodynamic effect

## Abstract

Gene therapy relying on the efficiency of gene delivery or transfection has become a widely applied approach in numerous biomedical fields. Ultrasound, featuring non-invasive and deep tissue penetration nature, offers effective enhancement treatment with precise targeting and minimal side effects. Here, we critically analyze the vectors, methods, and biomedical applications of ultrasound-enhanced gene transfection. Firstly, the therapeutic potential of ultrasound lies in its synergistic thermal, cavitation, and mechanical effects. Secondly, various vectors, both viral and non-viral, are available for delivering therapeutic genes. Additionally, techniques like sonoporation, sonodynamic effect, and ultrasound-targeted microbubble destruction enable the precise delivery of bioactive genes to targeted tissues. We particularly highlight the diverse biomedical applications of ultrasound-enhanced gene transfection across different disease areas, including cancer, cardiac insufficiency, stroke, neurodegenerative disease, and musculoskeletal disorders, demonstrating promising prospects for clinical application. Finally, we systematically address the current challenges and perspectives to inform the design of ultrasound-enhanced gene transfection compared to conventional gene therapies. This review aims to guide new developments in ultrasound-enhanced gene transfection and contribute to its widespread utilization in biomedical applications.

## Highlights

• Green extraction from *A. membranaceus* stem and leaf waste unlocks numerous bioactive flavonoids.
• Optimized surfactant-ultrasound extraction boosts antioxidant activity.
• Valorization of *A. membranaceus* stem and leaf waste can be effective way to attain sustainability.

## Graphical abstract

**Figure:**
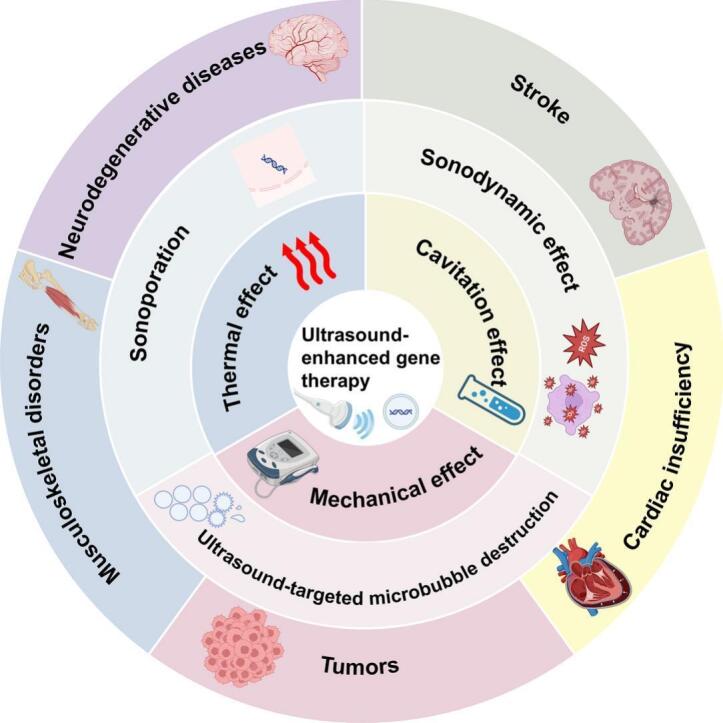

## 1 Introduction

Ultrasound refers to sound waves with frequencies greater than 20 kHz (above the human hearing threshold) with unique physical characteristics such as reflection, refraction, scattering, attenuation, absorption, and the Doppler effect [1,2]. By leveraging these properties and the acoustic characteristics of human tissue, ultrasound serves both diagnostic imaging and therapeutic functions [3,4]. Ultrasound can effectively penetrate soft tissues, providing high spatial resolution for real-time imaging, which is particularly valuable for guiding biopsies and therapeutic interventions [[5], [6], [7], [8]]. Additionally, ultrasound’s localized energy deposition enables targeted intervention of lesions [9,10].

Gene therapy emerged in the 1960 s and early 1970 s, which consists of transferring genetic material to patients to treat diseases [11] by overexpressing beneficial genes or suppressing harmful ones using gene editing tools [12]. Currently, gene therapy has become a widely applied approach in numerous therapeutic fields [[13], [14], [15]]. The latest advancements in molecular medicine have driven the creation of more targeted and efficient gene transfer vectors [16,17]. Current research on gene delivery vectors focuses on two main classifications: viral [18] and non-viral vectors [19]. Viral vector strategies are mainly based on lentiviruses [20], adenoviruses [21], and adeno-associated viruses [22]. Non-viral strategies for gene delivery include techniques such as electroporation [23], laser irradiation [24], microinjection [25], ultrasound-mediated transfection [26], polymers [27], lipids [28], inorganic particles [29], nanoparticles [30], enhance cell membrane permeability, facilitating the efficient delivery of gene material [31,32].

Gene transfection can be broadly categorized into several methods, including electroporation [33], acoustothermal transfection [34], laser irradiation [35], microinjection [36], magnetofection [37], and ultrasound-mediated transfection [38]. These methods facilitate the delivery of genetic material by inducing pores in cell membranes [[39], [40], [41]]. Ultrasound-mediated gene transfection offers a unique, noninvasive, and spatiotemporally controllable strategy with significant potential for biomedical applications [42,43]. Microbubbles, widely applied as ultrasound contrast agents, play a pivotal role in this process. Under ultrasound excitation, microbubbles not only allow real-time monitoring of tissue perfusion but also enable site-specific delivery of therapeutic agents and genes [44,45]. Ultrasound-targeted microbubble destruction (UTMD) facilitates greater permeability of microvascular walls, enhancing gene extravasation and delivery to target tissues [46]. Recently, the sonodynamic effect, where low-intensity ultrasound interacts with sonosensitizers to produce reactive oxygen species (ROS) through intracellular responses, has emerged as a potential strategy for gene transfection [[47], [48], [49], [50]].

Moreover, ultrasound can be integrated with many conventional modalities to enhance therapeutic outcomes. Gene therapy mediated by ultrasound has demonstrated considerable potential in addressing cancers, cardiovascular disorders, central nervous system diseases, and musculoskeletal conditions, with excellent clinical prospects [[51], [52], [53], [54], [55]]. In this review, we examine the advancements in gene therapy, the principal mechanisms of ultrasound-enhanced gene therapy, and their associated biological effects. We place special emphasis on the convergence of gene therapy and ultrasound, exploring the progress in targeted therapies aimed at improving treatment efficiency and specificity while also addressing some persistent challenges.

## 2 Biological effects of ultrasound

Ultrasound exerts biological effects primarily encompass thermal, cavitation, and mechanical phenomena. These effects have wide-ranging applications across the biomedical field, including ultrasound therapy, diagnostic imaging, and other clinical uses.

### 2.1 Thermal effect

Ultrasound’s thermal effects result from the conversion of acoustic energy into heat as it propagates within a medium. This conversion is due to the medium's internal friction and absorption, which result in a temperature increase. In this context, metalenses leverage their unique thermal effect to focus ultrasound waves with subwavelength resolution, enabling a significant temperature rise at low input energies that results in precise and efficient tissue ablation [56].

**Fig. 1:**
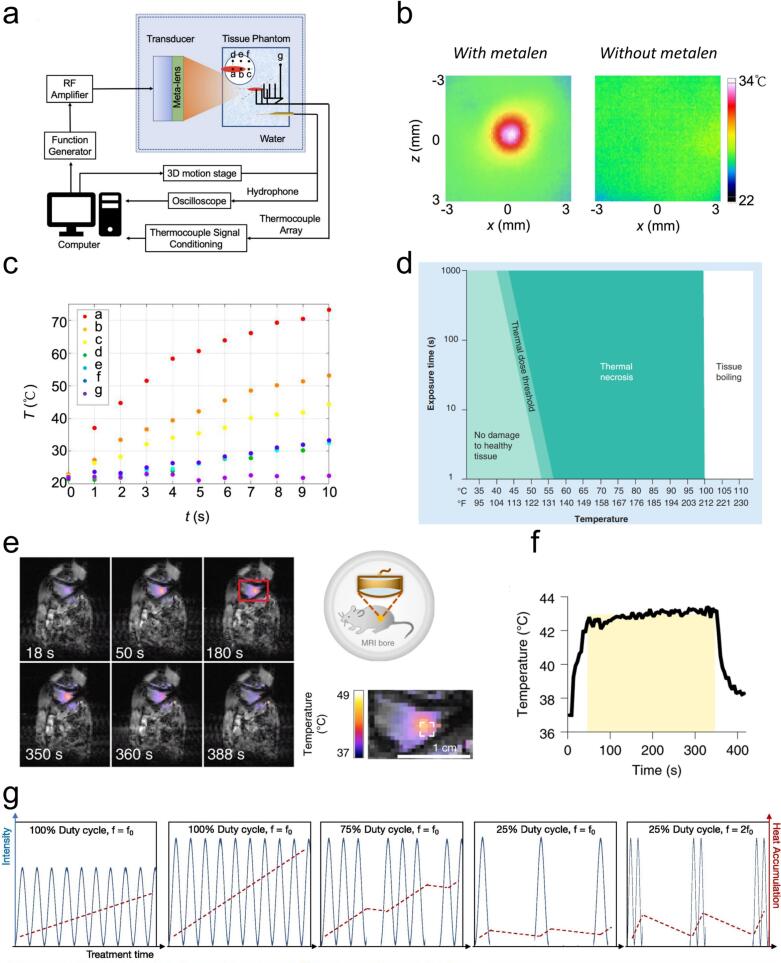
Thermal effect. a) Schematic diagram of 3D ultrasonic field scanning and temperature monitoring experimental device. b) Infrared thermal imaging, experimentally measured at the focal plane of the biological model’s surface, was used to visualize the temperature field with (left) and without (right) metalens. c) Temperature curves obtained from 7 thermocouple pins at different locations when using metalens [56]. Copyright 2023, Elsevier. d) Relationship between thermal dose and tissue biological effect [62]. Copyright 2013, Future Science Group. e) During 5-minute FUS stimulation of the hind limbs of anesthetized mice, color-coded temperature maps were overlaid on MRI scans at various time points. f) Mean temperature within the targeted region during FUS stimulation shown in panel e [63]. Copyright 2021, Springer Nature. g) The temperature increase of the thermal effect produced by ultrasound varies with the sound intensity, duty cycle, treatment time, and ultrasonic frequency [64]. Copyright 2021, Elsevier B.V.

Fig. 1a shows a hydrophone mounted on a three-dimensional motion platform used to map the ultrasound field following the metalens. Infrared thermography of the biological model’s surface at the focal plane was used to visualize the temperature distribution. The distinct focusing profile observed in the temperature distribution allows visualization of the thermal effects on the biological model (Fig. 1b). The temperature profiles recorded at different locations are shown in Fig. 1c. When ultrasonic energy is continuously transmitted, a portion is absorbed and transformed into heat, which raises temperature of the tissue at the focal point. The magnitude and duration of this temperature increase are quantified as the tissue’s “thermal dose” (Fig. 1d). Tissue remains undamaged below the thermal dose threshold, while exceeding this threshold leads to tissue necrosis. In focused ultrasound therapy, higher thermal doses accelerate tissue necrosis. Focused ultrasound (FUS) can safely and non-invasively deliver energy to tissues several centimeters deep. MRI-guided FUS allows precise deposition of thermal energy into localized areas of the body with high spatiotemporal resolution. In vivo studies using MRI-guided FUS showed controlled local heating, as indicated in mice with Nalm-6 cells carrying dual luciferase reporter genes. The FUS pulses induced a significant increase in fluorescence intensity, indicating robust gene expression activation (Figs. 1 e–f). The local heat generation is determined by the intensity and frequency of ultrasound waves, as well as the tissue’s absorption coefficient. At a fixed duty cycle, higher intensity results in greater heat buildup (Fig. 1g). High levels of ultrasound energy deposition can cause a dramatic temperature rise, leading to thermocoagulation and cell death. Consequently, most clinical applications of FUS currently under investigation rely on thermal mechanisms for deep tissue ablation [57,58].

Hyperthermia exhibits a biphasic effect on tumor physiology. At lower temperatures, the thermal effect enhances perfusion and increases capillary permeability, facilitating the influx of drugs or gene carriers. However, higher temperatures can result in vascular damage and increased hypoxia [59,60]. Moreover, hyperthermia can selectively enhance drug delivery and release rate from thermosensitive liposomes, potentially enabling targeted tumor therapy [61].

### 2.2 Cavitation effect

**Fig. 2:**
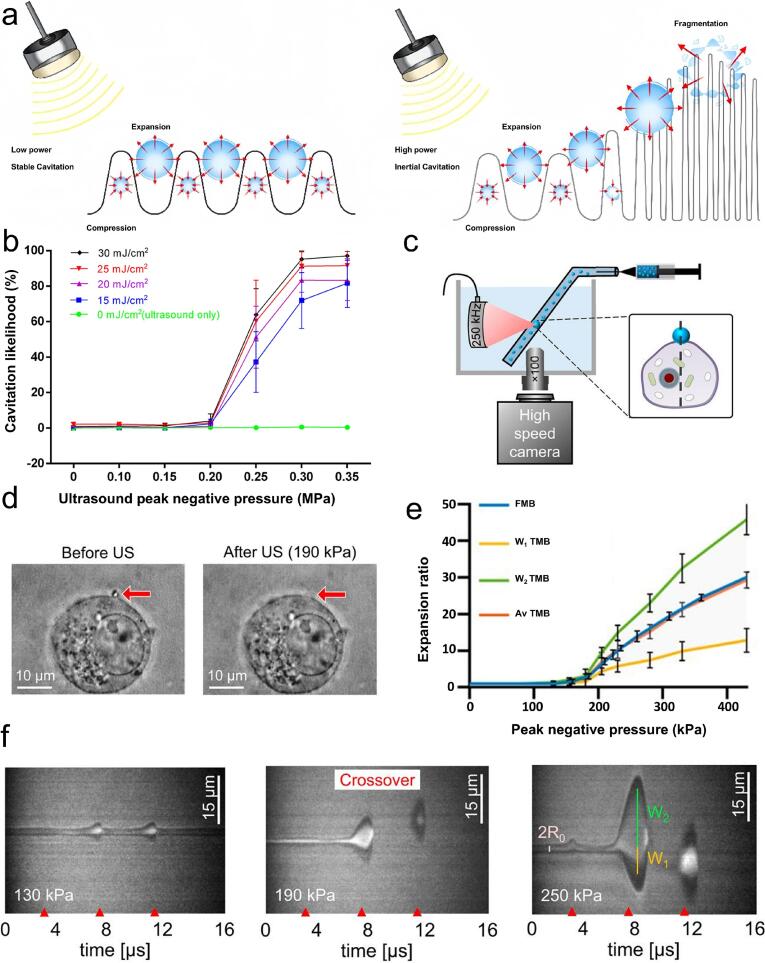
Cavitation effect. a) Interaction between microbubbles and ultrasonic waves. Non-inertial cavitation (left) and inertial cavitation (right) [78]. Copyright 2023, Ivyspring International Publisher. b) The cavitation possibility of light-mediated ultrasound during PUT was studied by using B-mode ultrasound imaging system in conjunction with a passive cavitation detector [79]. Copyright 2017, Springer Nature. c) Ultrahigh speed imaging device. d) After US pulse emission subjected to a 190 kPa peak negative pressure (PNP), the targeted microbubble (TMB) is disrupted. e) The expansion rate of TMBs as a function of PNP, the bounded wall (W1) and free wall (W2) of microbubbles, as well as the average (Av) expansion rate. f) Stable cavitation was observed at 130 kPa, a transition to inertial cavitation occurred at 190 kPa, and inertial cavitation dominated at 250 kPa [70]. Copyright 2020, National Academy of Sciences.

Ultrasonic cavitation occurs when microscopic bubbles (cavitation nuclei) within a liquid oscillate, expand, and absorb acoustic energy in response to ultrasonic waves. Once the energy surpasses a specific threshold, the cavitation bubbles collapse violently and implode, generating intense localized energy. This process involves dynamic changes, including the formation, oscillation, growth, and rupture of bubbles. Cavitation is a complex mechanism that describes the behavior of gas-filled bubbles under ultrasonic irradiation and is considered the primary driving force for promoting targeted delivery [65]. In fluids, cavitation bubbles driven by pressure exhibit a nonlinear behavior, releasing extremely high energy densities as they collapse. When cavitation arises adjacent to a rigid surface, bubbles commonly collapse unevenly, producing high-speed liquid jets capable of inducing localized damage. Since encapsulated microbubbles (MBs) are frequently employed to boost echogenicity in ultrasonic diagnostic imaging, this cavitation can also result in jet-induced tissue damage [66]. Both low- and high-intensity ultrasound can induce the expansion and contraction of gas nuclei, giving rise to two primary cavitation regimes, stable and inertial cavitation, depending on the type of MBs used (Fig. 2a). Cavitation behavior is influenced by multiple factors, such as frequency, pressure amplitude, bubble radius, and the surrounding environment [67,68].

The possibility of cavitation can be quantified using a passive cavitation detector (PCD). For instance, in the absence of laser illumination, when using ultrasound alone, or with peak negative ultrasound pressure below 0.2 MPa, the probability of cavitation was nearly zero. At higher laser energy densities, ranging from 15 to 30 mJ/cm^2^, significant cavitation was observed only when the peak negative ultrasonic pressure exceeded 0.2 MPa. The cavitation probability reached saturation once the peak ultrasonic negative pressure surpassed 0.3 MPa (Fig. 2b).

Ultrahigh-speed imaging was employed to capture the oscillation of target microbubble (TMB) adhering to cells. The TMB attaches to the top of the cell and is destroyed following the application of an ultrasound pulse delivering a peak negative pressure (PNP) of 190 kPa. Different cavitation states are induced under varying PNP levels. As the PNP increases, the microbubbles transition from stable to inertial cavitation (Fig. 2 c–f). Stable cavitation describes the sustained growth and oscillation of cavitation bubbles over several acoustic cycles. In the presence of MBs, repeated contraction and expansion at low sound pressure leads to stable cavitation, generating fluid flow and microbubble wall deformation. This process causes membrane perturbation, which enhances endocytosis and promotes the transport of macromolecules, leading to the stimulation of ion channels and receptors. There may also be secondary effects on cell permeability and electrical activity, which, in some cases, can result in membrane rupture [[68], [69], [70]]. In contrast, inertial cavitation arises when ultrasound induces elevated pressure amplitudes, leading to MBs’ rapid growth and immediate collapse. This type of cavitation causes the bubbles to implode, generating microjets and shock waves that perforate the cell membrane, promoting its permeability and facilitating the entry of therapeutic agents into the extravascular space [[71], [72], [73]]. Ultrasound combined with MBs promotes the creation of temporary pores in the plasma membrane through cavitation. In the presence of MBs, ultrasound-induced cavitation plays a dual role in the localized release of drugs from their carriers while enhancing plasma membrane permeability. These collective effects facilitate targeted cellular uptake or enable the entry of substances that would otherwise be impermeable [69,74,75].

When the negative pressure amplitude surpasses the medium’s intrinsic threshold, a single pulse containing a strong negative phase can produce a cavitation cloud, giving rise to histotripsy. This process relies on initiating a cavitation cloud to segment soft tissue, making non-invasive, focused ultrasound treatment for tissue ablation possible. Unlike thermal ablation, which uses heat for therapeutic ultrasound, histotripsy-based tissue biopsy relies on the mechanical action of the cluster of cavitation bubbles to destroy tissue. Although the activity of sound bubbles is often described as chaotic, brief tissue bursts generate a distinct and reproducible form of cavitation that is effective for controlled tissue ablation [76,77].

### 2.3 Mechanical effect

When ultrasound waves propagate through biological tissues, the sound pressure induces various biomechanical changes, including material vibration, volume alterations, cytoplasmic flow, and the oscillation and rotation of cytoplasmic particles. These changes result in two types of mechanical effects. The first effect is in the traveling wave field, which affects cell membranes permeability, accelerates blood and lymphatic circulation, and facilitates other physiological processes. The second effect is in the standing wave field, inducing material diffusion within the tissue cells through ultrasonic vibrations. This stimulation of the diffusion process across the semi-permeable cell membrane promotes cell regeneration. These processes enhance metabolism, improve tissue nutrition, and support the cells' ability to regenerate and repair [[80], [81], [82]].

## 3 Gene delivery vectors

**Fig. 3:**
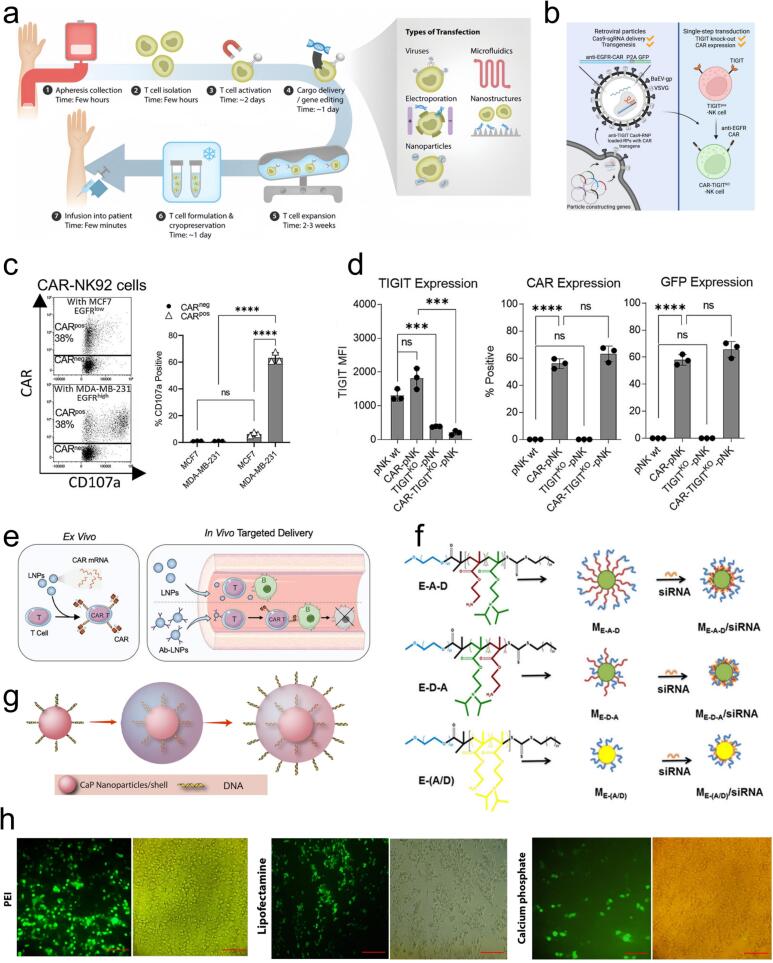
a) Fabrication and transfection of chimeric antigen receptor (CAR)-T cells. CAR-T cells can be engineered through various transfection methods, including viral vectors, electroporation, microfluidics, nanoparticles, and high-aspect-ratio nanostructures [103]. Copyright 2021, Wiley‐VCH GmbH. b) Engineered natural killer (NK) cells were knocked out by retroviral particle (RP) with anti-EGFR (epidermal growth factor receptor)-CAR transgene and TIGIT gene. c) Exemplary dot and bar plot illustrating the enhanced anti-tumor activity of CAR-NK 92 cells against MDA-MB-231 high TNBC cell line with EGFR. d) Expression of TIGIT, CAR, and GFP (green fluorescent protein) was evaluated on primary NK cells derived from three different donors, following transduction with RP [104]. Copyright 2023, Elsevier. e) CAR T cells were produced by transfecting T cells with ionizable lipid nanoparticles (LNPs) in vivo and in vitro [92]. Copyright 2023, Wiley‐VCH GmbH. f) Molecular structure of E–A–D, E–D–A, and E−(A/D) polymers [95]. Copyright 2017, American Chemical Society. g) Schematic illustration of triple-shell calcium phosphate (CaP) nanoparticles incorporating DNA [105]. Copyright 2020, Elsevier B.V. h) Transfection efficiency in HEK-293 T cells was compared using three methods—PEI, liposome-based transfection, and calcium phosphate—by assessing GFP expression [106]. Copyright 2021, Springer Nature.

For the therapeutic applications in human diseases, traditional small-molecule drugs and antibody-based therapies typically target downstream proteins associated with gene-related conditions. Over the last twenty years, gene therapy has developed into a highly precise and efficient therapeutic approach [83,84]. Various methods are currently available for delivering therapeutic genes, including viral and non-viral delivery systems (Fig. 3a). These approaches enable the modification of host cell genetic content, facilitating extensive studies of normal cellular processes, the molecular mechanisms underlying diseases, and the therapeutic potential of gene-based interventions [85]. Different delivery strategies have their own advantages and limitations in clinical applications. Table 1 compares the common gene delivery methods and summarizes the key factors that need to be considered in their practical applications.

**Table 1:** Summary of the efficiency and limitations of common gene delivery strategies.

Gene delivery vectors	Capacity packing	Transfection efficiency	Immunogenicity	Target ability	Duration of gene expression	Main advantage	Main limitations	References
Adenovirus (AdV)	high	high	high	medium	short	Highly efficient transduction of dividing/non-dividing cells	Strong immune response, prone to cause inflammation	[86,87]
Adeno-associated virus (AAV)	low	high (depending on the serotype)	low	high (multiple serotypes)	long	High safety and long-term expression	Small packaging capacity	[88,89]
Lentivirus (LV）	medium	high	medium	medium	long	Infect non-dividing cells and stably integrate	insertion mutation risk and high production cost	[13,90]
Lipid nanoparticle vectors	high	medium to high	low to medium	low(modifiable)	short	High safety, easy to produce and large capacity	Transfection efficiency varies by cell type	[91,92]
Polymer vectors	high	low to medium	low to medium	low(modifiable)	short	Flexible design and easy to produce	Low transfection efficiency and may be cytotoxic	[[93], [94], [95]]
Calcium phosphate vector	high	low to medium (higher in vitro, limited in vivo)	very low	low (passive targeting)	short	Good biocompatibility, degradable, PH-responsive release (lysosomal escape), and low cost	Poor stability in the body, difficult to store, prone to aggregation, and complex conditions for optimization	[[96], [97], [98]]
Ultrasound-mediated gene delivery	unlimited	low to high	low	high (physical space targeting)	short	Non-invasive or minimally invasive, excellent spatial targeting, capable of deep tissue penetration, combined with microbubbles to enhance efficiency	Efficiency is sensitive to parameters, may cause local tissue damage, and usually requires the combination of microbubbles or carriers	[99,100]
Electroporation	unlimited	high	low	low	short	Straightforward method and applicable to multiple cell types	High cell mortality rate, mainly apply in vitro	[101,102]

### 3.1 Viral delivery vectors

Viral vectors facilitate the delivery of targeted nucleic acid into host cells in viral-mediated transfection, making it an effective strategy for transfecting cells that are challenging to transfect using conventional methods [107]. Contemporary viral vector-based gene therapy utilizes vectors derived from retroviruses (such as lentiviruses) [90], adenoviruses [87], or adensao-associated viruses [108], among others, to induce an immunogenetic response in host cells. Lentiviral vectors, derived from specific species of lentiviruses within the retrovirus family, have become crucial tools in gene therapy. These vectors, particularly regulatable lentiviral systems, have been extensively employed in basic research to investigate gene functions or to transiently reprogram cells [90]. Research indicates that retrovirus-mediated appproaches enable both CRISPR-Cas9 (clustered regularly interspaced short palindromic repeats-associated protein 9) gene knockout and CAR transgene insertion in NK cells. This strategy enables the generation of NK cells expressing anti-epidermal growth factor receptor (EGFR)-CAR, while simultaneously knocking out the TIGIT gene. This strategy holds promise for enhancing the effectiveness of NK cell-based immunotherapy (Fig. 3 b–d). Adenoviral vectors are favored as gene delivery vehicles because of their stability, capacity of large gene transfer, high titer production, and exceptional transduction efficiency [86,87]. Adeno-associated viruses (AAV) have become prominent vectors because of their numerous desirable properties, including non-pathogenicity, the capacity to infect both proliferating and quiescent cells, and continuous maintenance of the viral genome within host cells [88,109]. In addition to their role in gene delivery, viral vectors can also enable the production of therapeutic proteins within the host cells. Self-replicating RNA viruses, for instance, provide significant RNA amplification in the cytoplasm, thereby enhancing gene silencing efficacy [110]. Peptide nanofibrils, which bind to negatively charged virions, enhance viral transduction by facilitating active engagement with cellular protrusions, thereby promoting virion attachment and increasing cellular entry and gene transfer efficiency [111]. However, despite these advantages, viral transduction is plagued by several limitations, including potential cytotoxicity, varied tropism, difficulties in targeting specific tissues, limited gene delivery capacity, and the risk of inducing carcinogenesis and insertional mutagenesis. These persistent challenges hinder the full potential of viral vectors in therapeutic application [13].

### 3.2 Lipid nanoparticle vectors

The lipid nanoparticle (LNP)-based delivery platform is considered among the most sophisticated and effective non-viral carrier for therapeutic applications across a broad spectrum of diseases. LNPs consist of phospholipids, ionizable lipids, cationic lipids, cholesterol, and polyethylene glycol (PEG) lipids. Upon reaching the acidic endosome of a cell, the ionizable lipids undergo ionization, facilitating endosomal escape and subsequent cytoplasm delivery of the genetic cargo, thereby enhancing therapeutic effect. Conversely, cationic polymer-based mRNA nanoparticles show limited internalization and low transfection efficiency in natural killer (NK) cells. In contrast, encapsulating mRNA with LNP (mRNA-LNP) and optimizing both lipid composition and microfluidic manufacturing conditions markedly enhances NK cells transfection rates and elevates protein expression [91]. In vitro engineering of CAR T cells is both costly and technically demanding. Nevertheless, LNP-mediated CAR mRNA delivery provides a more efficient alternative, reducing off-target effects and improving targeting in both experimental and clinical contexts. Traditional LNP methods generate CAR T cells by transfecting patient-derived T cells in vitro, whereas antibody-conjugated (Ab)-LNP enable direct in vivo programming of T cells, thereby streamlining CAR T cell generation (Fig. 3e).

The ocular delivery of mRNA-LNPs enables efficient retinal gene delivery and editing capabilities, potentially correcting genetic mutations contributing to blindness [112]. Additionally, non-liposomal lipid nanovesicles conjugated with miRNA represent an effective strategy for transporting genetic material into tumor cells cytoplasm, where they can elicit tumor-suppressive effects. Beyond oncology, this strategy also holds promise for the therapeutic administration of miRNAs and other small regulatory RNAs, thereby paving their way for clinical applications [113]. However, a significant challenge with LNPs is their rapid clearance from the bloodstream in vivo, which triggers a strong inflammatory response and results in high toxicity levels, significantly limiting their broader application.

### 3.3 Polymer vectors

Polymers provide a solid foundation for novel mRNA delivery systems, particularly for effective T-cell transfection [93]. Among these, charge-changing releasable transporters (CARTs) are distinguished by their beta-amino carbonate (bAC) backbone and unique side chain arrangement, which has shown enhanced mRNA delivery capabilities. These bAC-CARTs significantly improved mRNA transfection efficiency in Jurkat cells and demonstrated superior protein expression in vivo, thanks to their improved bAC polymer structure [114]. Furthermore, biodegradable poly (beta-amino ester) (PBAE) polymers are extensively utilized as non-viral delivery vehicles for in vitro-transcribed (IVT) mRNA that codes for antigen-specific receptors. In situ genetic reprogramming of human T lymphocytes is achieved using PBAE nanoparticles to encapsulate and transport this IVT mRNA. This technique facilitates the transient expression of therapeutic receptors, including chimeric antigen receptors (CARs) and T cell receptors (TCRs), that are engineered to target disease-specific antigens, therby enabling the rapid generation of engineered cell functions without genomic integration [115]. Similarly, biodegradable poly(amine-co-ester) (PACE) polyplexes, as inhalable polymer-based vehicles, have achieved high transfection efficiencies in pulmonary epithelial and antigen-presenting cells, achieving high expression of therapeutic mRNAs to the lung [91,94]. Despite their promising prospects, polymer-based carriers face significant challenges, including low encapsulation efficiency, cation-induced cytotoxicity, and destabilization from negatively charged biomacromolecules, impeding their clinical application. These limitations can be addressed by improving self-assembly properties and stability through hydrophobic interactions and PEGylation (Fig. 3f). While polymers are a promising avenue for mRNA delivery, their cellular uptake, organotropic specificity, and efficacy in hard-to-transfect cells remain opportunities for further research and breakthroughs.

### 3.4 Calcium phosphate vector

Calcium phosphate (CaP) nanoparticles are a promising class of non-viral gene vectors, offering advantages such as low toxicity, facile synthesis, and high transfection efficiency. The crystalline structure of CaP exhibits a strong affinity for nucleic acids and high biocompatibility, enabling the delivery of therapeutic materials, including nucleic acids (Fig. 3g), small-molecule drugs, proteins, and enzymes, to tumor cells. Studies indicate that the physicochemistry properties of CaP nanoparticles—such as particle size, surface charge, morphology, chemical composition, and surface functionalization—play a pivotal role in dictating their cellular uptake mechanisms. These parameters critically influence cellular internalization, determining their effectiveness in nanomedicine applications [96,116,117]. However, their clinical application has been hindered by the uncontrolled and rapid growth of CaP crystals. These challenges can be addressed by employing nanoscale mixed nanoparticles composed of CaP and PEG-stabilized polyanionic block copolymers. Such hybrid systems enhance colloidal and serum stability, reduce nonspecific interactions, and exhibit markedly improved gene silencing effects relative to conventional CaP/siRNA nanoparticles [97,118]. Within these nanocomposites, the interaction of PEG-b-poly(benzoxaborole) (PEG-PBO) with both siRNA and CaP enhances siRNA loading capacity and stabilizes the nanoparticle structure. Furthermore, pH-responsive PEG-PBO/siRNA/CaP hybrid nanocomposites have proven efficiently in delivering siRNA to various cancer cells, showcasing their potential in cancer therapy and other applications [98].

## 4 Ultrasound-enhanced gene transfection methods

Extensive research has investigated the cellular mechanisms of ultrasound-mediated gene delivery, revealing insights into its efficiency and underlying processes [99]. Using ultrasound in conjunction with ultrasound-sensitive particles enables the precise and site-specific transfer of genetic materials to target organs or tissues. This approach not only increases cell membrane permeability but also enhances gene uptake [65]. For instance, focused ultrasound (FUS) therapy combined with cationic ultrasound contrast agents (UCAs) has demonstrated tumor-specific transfection of plasmid DNA (pDNA) encoding the inducible nitric oxide synthase (iNOS) enzyme. This strategy resulted in significant improvements in tumoral perfusion, potentiated the efficacy of chemotherapeutic agents, and extended survival in an orthotopic xenograft model [119]. Furthermore, the application of an ultrasound-sensitive mannose-modified gene delivery system alongside doxorubicin-loaded polyethylene-glycol (PEG)-modified liposomes has been shown to inhibit early-stage tumor progression effectively. This combination further enhanced transfection efficiency in antigen-presenting cells, thereby boosting the clinical promise of DNA-based vaccination strategies [100]. Additionally, therapeutic ultrasound (TUS) treatments with the human tumor-suppressive gene hSef-b demonstrated significant inhibition of prostate tumor growth. The hSef-b plasmid suppressed cell proliferation, downregulated the in vivo levels of proangiogenic factors FGF2 and MMP-9, and consequently reduced blood vessel density. These findings highlight the therapeutic effectiveness of a non-viral TUS-mediated hSef-b gene delivery platform as a potential therapeutic strategy for the management of prostate cancer [120].

### 4.1 Sonoporation

**Fig. 4:**
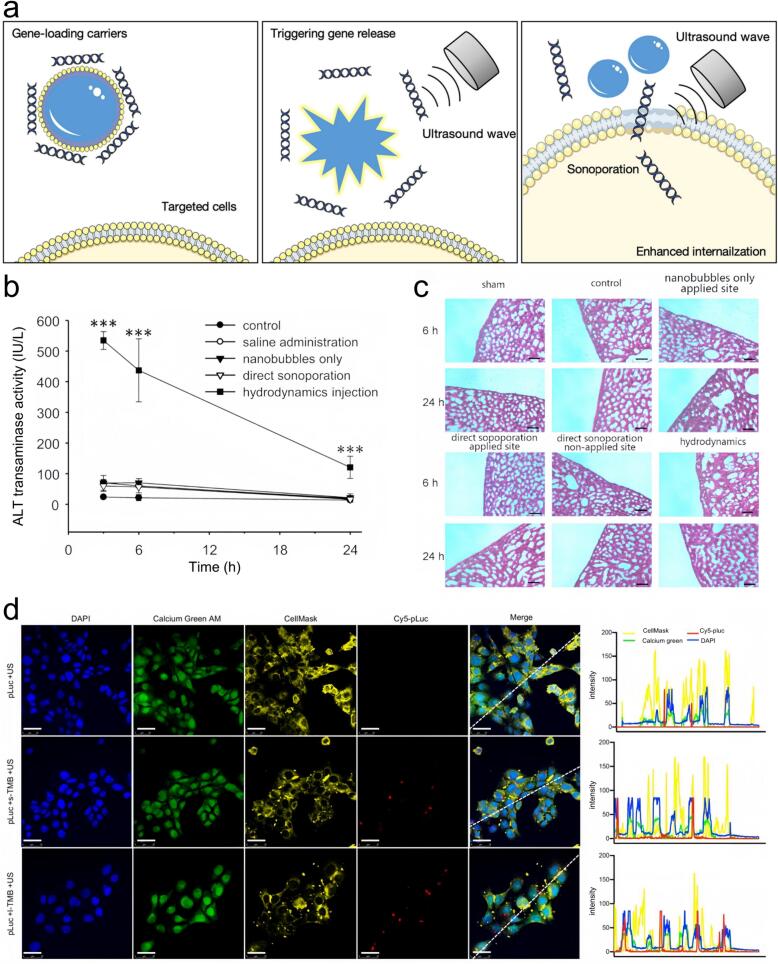
a) Ultrasound triggers gene release from the carrier and enhances gene internalization through sonoporation [64]. Copyright 2021, Elsevier. b) Assessment of liver injury after surface-direct sonoporation included measurements of serum alanine aminotransferase (ALT) and histopathological evaluation by H&E staining (c) [126]. Copyright 2019, MDPI. d) Confocal laser scanning microscopy illustrate the spatial distribution of Cy5-pLuc following acoustic perforation under three conditions: without targeted microbubble (TMB) (top), with small targeted microbubble (s-TMB) (middle), and with large targeted microbubble (l-TMB) (bottom). Fluorescence intensity along the dotted lines in each channel of the microscopic images was quantified for spatial analysis [142]. Copyright 2021, Wiley‐VCH GmbH.

Sonoporation refers to the phenomenon in which ultrasonically activated ultrasound contrast agents (UCAs) oscillate near biological barriers, inducing transient membrane permeabilization, thereby facilitating the uptake of molecules [121]. Ultrasound combined with UCAs induces sonoporation, generating transient damage to the cell membrane. This process creates temporary and reversible pores, permitting the translocation of therapeutic molecules, including genes, via multiple pathways, including the cell membrane, endocytosis, and cell junctions [122] (Fig. 4a). Cells subjected to ultrasound in the combination with merocyanine 540 exhibited multiple surface pores. Observations showed dimple-like craters on the cell surface, accompanied by structure damaged in which cytoplasmic material appeared to extrude beyond the plasma membrane [123]. Subsequent studies validated that ultrasound, either applied independently or in combination with microbubbles (MBs), enhances the permeability of the plasma membrane through sonoporation, enabling extracellular substances to enter the cell [124,125]. Sonoporation has shown promise in enabling site-specific transfection into targeted areas, such as the brain, liver, and kidney, due to cavitation-induced mechanical forces that generate temporary pores on cellular membranes. A “direct sonoporation” system has been developed to facilitate gene transfer restricted to a defined peritoneal region, minimizing unintended systemic exposure. This site-specific transfection method targets peritoneal mesothelial cells without inducing transgene expression elsewhere, and it exhibits minimal toxicity in adjacent tissues via cavitation energy. This approach has potential applications in developing intraperitoneal sonoporation devices for treating peritoneal diseases like peritoneal fibrosis [126,127] (Fig. 4 b–c). Sonoporation also provides a potential reversible membrane and cell wall penetration method for plant cells, which has significant application value in the fields of gene transfection and plant biotechnology. Studies have shown that by using ultrasound-mediated methods, it is possible to assist polyethyleneimine-coated mesoporous silica nanoparticles in efficiently introducing exogenous genes into suspension cultured plant cells, constituting an innovative method for secure and controlled gene transfer. The greatest advantage of this method lies in its strong versatility, which can overcome the dependence of traditional agrobacterium method and gene gun method on plant species and genotypes, and is especially suitable for crops that are difficult to transform. In addition, ultrasonic treatment itself has a certain biological stimulating effect on cells, which can promote cell division and metabolic activity, and create a more favorable microenvironment for the expression of exogenous genes [128,129]. Alongside its role in cell membrane permeabilization, sonoporation can transiently augment vascular permeability, supporting efficient transport of drugs into surrounding tissues. The vascular endothelium constitutes a critical barrier for systemically administered drugs, as successful therapy depends on their extravasation to reach target tissues [130,131]. Physiological barriers, such as tight junctions, hinder the efficient delivery of drugs to their therapeutic targets. Sonoporation enhances endothelial permeability, thereby improving therapeutic delivery efficiency [132]. Focused ultrasound (FUS) transiently increases vascular permeability via mechanical and thermal mechanisms, enabling the more efficient extravasation and diffusion of therapeutic agents into surrounding tissues. FUS combined with MBs alleviates vascular barriers, enhances interstitial convective transport by increasing hydraulic conductivity, and significantly improves endothelial uptake of small therapeutic agents [133,134]. Sonoporation is governed by a shear-stress threshold induced by microbubble oscilation, typically on the order of kilopascals, causing enhanced permeability of the endothelial barrier. This shear-stress threshold exhibits an inverse square root relationship with the number of oscillation cycles and varies linearly with ultrasound frequencies in the 0.5–2 MHz range. Real-time microscopic observations have elucidated both the cavitation-driven phsical mechanisms of sonoporation and the membrane-mediated biophysical process through which microbubble oscillations acutely and durably enhance permeability of cells and vasculature [135].

The efficacy of tumor therapy can also be improved via vaporization-enhanced sonoporation mediated by gold nanodroplets. Studies have shown that sonoporation rates increase during the rarefaction phase, upon exposure to a pulsed wave (PW) laser. This approach offers the potential to improve the efficacy of drug delivery and tumor treatment using reduced laser power, while preserving cell viability and minimizing cytotoxic effects [136]. Recent insights into the cellular and molecular basis of human diseases indicate that sonoporation represents a promising non-viral strategy for gene therapy, offering targeted and controllable delivery of gene material [123,137]. Sonoporation integrates the targeted application of ultrasonic waves with the intravascular or intratissue delivery of gaseous MBs, transiently increasing vessel and tissue permeability for otherwise poorly permeant molecules [138]. Ultrasound excitation of MBs targeted to HEK-293 cell membranes has been shown to induce membrane permeabilization that is precisely controlled in both space and time, with high reproducibility across repeated applications. By combining patch clamp electrophysiology with fluorescence imaging, researchers characterized pre formation and resealing dynamics, achieving fine control of ultrasound-induced membrane permeabilization in individual cells. Additionally, ultrasound application can noninvasively target specific tissue volumes in vivo, positioning sonoporation as a powerful and adaptable platform for nonviral therapeutic delivery [139]. A novel application of sonoporation facilitates intracellular delivery of fluorescent dyes, enabling real-time visualization and quantitative assessment of gap junction intercellular communication (GJIC) in human embryonic stem cell (hESC) colonies. Transient membrane pores can be induced by delivering brief ultrasound pulses that selectively excite individual microbubbles (MBs) anchored to the cell membrane, allowing intracellular dye loading and calcium influx into single hESCs [140]. While many studies on sonoporation involve strong ultrasonic fields causing multiple simultaneous phenomena, mild (linear) oscillations of microbubbles have been shown to induce precise disruption of lipid membranes without causing extensive collateral damage. Such controlled methodology permits applications ranging from cellular manipulation and cell wall permeation to microfluidic device implementation [141]. To investigate the impacet of MB-enhanced sonoporation's on DNA transfection, Cy5-labeled luciferase plasmid (Cy5-pLuc) was employed as a gene delivery reporter in cells cultured on coverslips. Confocal microscopy demonstrated that TMB-induced intracellular transport of Cy5-pLuc through transient membrane pores led to increased Cy5 fluorescence within both the cytoplasm and nucleus, indicating enhanced nuclear uptake ang cytosolic distribution. The pore size estimated from fluorescence intensity measurements, showed that l-TMBs and ultrasound induced larger pores, confirmed by using DAPI, calcium green AM, CellMask, and Cy5 fluorescence (Fig. 4d).

### 4.2 Sonodynamic effect

The sonodynamic effect refers to the use of ultrasonic waves to activate specific chemical agents, known as acoustic sensitizers, which then trigger a series of biochemical reactions in cells or tissues. This phenomenon has significant applications in the medical field, particularly in tumor therapy. The sonodynamic effect is primarily based on the activation of sonosensitizers by ultrasound energy, which subsequently produces singlet oxygen and other reactive oxygen species (ROS), which possess potent cytotoxic properties and are capable of inducing tumor cell death [143]. Acoustic sensitizers are typically characterized by simple chemical structures with good selectivity and preferential accumulation in tumor tissues. Their aromatic ring structures facilitate efficient photon energy transfer, with common examples including hematoporphyrin, pheophorbide A, the Ga–porphyrin complex ATX-70, merocyanine, dimethylformamide, erythrosine B, tenoxicam, and piroxicam [144]. ROS are highly reactive oxygen-containing molecules that can generate free radicals in living organisms. The asymmetric rupture of acoustic cavitation bubbles induces mechanical damage, enhancing membrane permeability and leading to erosion and wear. During cavitation, the gas and vapor within the bubbles are compressed, creating a localized hotspot with extremely high temperatures, which induces sonochemical effects in the surrounding medium. These effects facilitate reactions between water molecules and dissolved oxygen, increasing the local concentration of free radicals. These free radicals can react with various cellular components, leading to chemical changes that may result in cell death [145,146]. The energy delivered by ultrasound during sonodynamic therapy (SDT) is precisely focused on malignant tumors, activating acoustic sensitizers that preferentially accumulate in tumor tissues. This selective accumulation allows targeted tumor cell destruction simultaneously sparing adjacent healthy tissue from adverse effects. As a novel therapeutic strategy, SDT holds significant potential in cancer treatment, utilizing ultrasound in combination with acoustic sensitizers to induce tumor cell death. This technique has been explored as a potential adjunct to cancer immunotherapy, where it promotes cell surface calreticulin expression, elicits immune responses, induces functional antitumor vaccination, and can even induce the abscopal effect, all with fewer toxic side effects compared to conventional chemotherapy agents [[147], [148], [149]].

**Fig. 5:**
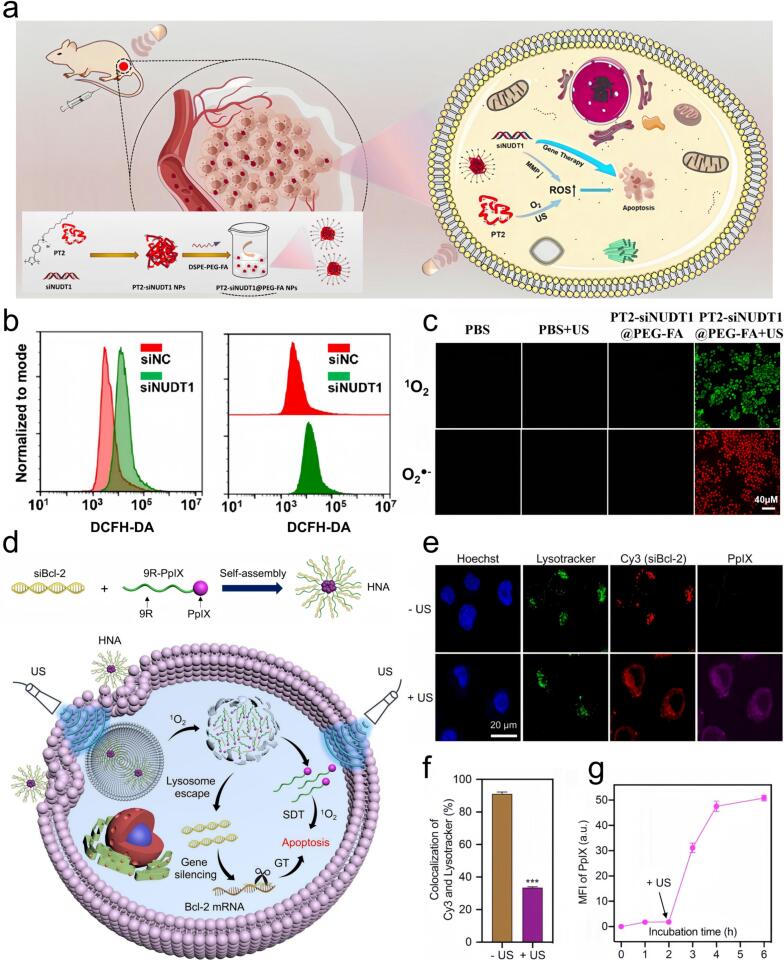
a) Illustrative schematic detailing the fabrication and therapeutic mechanism of novel tumor-targeting nanomedical cationic polythiophene derivative PT2-siNUDT1@PEG-FA NPs. b) ROS production was detected in HepG2 cells after transfection with si-NUDT1. c) ROS generation capacity of PT-2 fluorescent images of HepG2 cells of O22– and DHE stained with PBS, PBS + US, PT 2-siNUDT1@PEGFA NP, and PT 2-siNUDT 1@PEG-FA NP + US [153]. Copyright 2024, American Chemical Society. d) Schematic of hybrid nano assembly (HNA) design. After internalization of HNA, sequential ultrasound irradiation generates singlet oxygen, drives cytoplasmic siBcl-2 delivery to suppress Bcl-2 mRNA, and amplifies SDT efficacy. e) Confocal images showing cellular uptake and lysosomal escape of Cy3-HNA (300 nm) in HeLa cells after 4 h. Nuclei and lysosomes were respectively labeled with Hoechst and Lysotracker. f) Colocalization ratio of Cy3 and Lysotracker signals. g) MFI of PpIX in HeLa cells treated with Cy3-HNA (300 nm) from 0 to 6 h [154]. Copyright 2023, Elsevier B.V.

The past few years have seen notable progress in the use of SDT as a cancer treatment modality. For example, the application of tin monosulfide nanoparticles (SNSNPs) as nano-acoustic sensitizers has been demonstrated to penetrate the dense extracellular matrix of the tumor microenvironment, thereby enhancing the therapeutic efficacy of sonodynamic therapy (SDT) against triple-negative breast cancer (TNBC) while minimizing off-target effects [150]. In another study, Chlorin e6 and anti-PD-L1 antibody encapsulated within lipid nanobubbles (Ce6@aPD-L1 NBs), combined with ultrasound therapy, have effectively targeted prostate cancer. These Ce6@aPD-L1 NBs not only promote ROS generation but also enabled tumor-specific delivery, thereby improving the therapeutic efficacy of SDT [151]. Moreover, the cationic polythiophene derivative PT2, as a gene carrier for siRNA, has been utilized in SDT, with siNUDT1 inhibiting tumor cell proliferation and augmenting ROS production. As a result, a novel tumor-specific nanomedicine, PT2-siRNA-@PEG-FA, has been developed for synergistic SDT and liver cancer gene therapy (Fig. 5 a–c). Ultrasound has also been shown to enhance cytoplasmic siRNA delivery when combined with ultrasound gene therapy for cancer. Hybrid nano-assembly (HNA), formed via electrostatic self-assembly between siRNA and nona-arginine conjugated with protoporphyrin IX, generate singlet oxygen upon cellular uptake and ultrasound exposure. By facilitating intracellular transport of siBcl-2, this approach depletes Bcl-2 mRNA and amplifies the combined therapeutic efficacy of SDT (Fig. 5 d–g). For example, The sonosensitive bifidobacterium platform HMME@BiL harnesses hypoxic tumor tropism for SDT, and SR717, a STING pathway agonist, synergistically enhances immune responses, resulting in robust eradication of primary and metastatic tumors with excellent biocompatibility [53]. The management of pancreatic cancer, a highly aggressive malignancy, has been advanced through the synergistic use of SDT alongside other therapeutic strategies. For example, oxygen-carrying microbubbles (MBs) loaded with gemcitabine, combined with chemotherapy and SDT, have been proven effective for targeted pancreatic cancer therapy in mouse models [152]. These advancements underscore the feasibility of SDT for clinical applications and provide novel strategies for future cancer treatments.

### 4.3 Ultrasound-targeted microbubble destruction (UTMD)

**Fig. 6:**
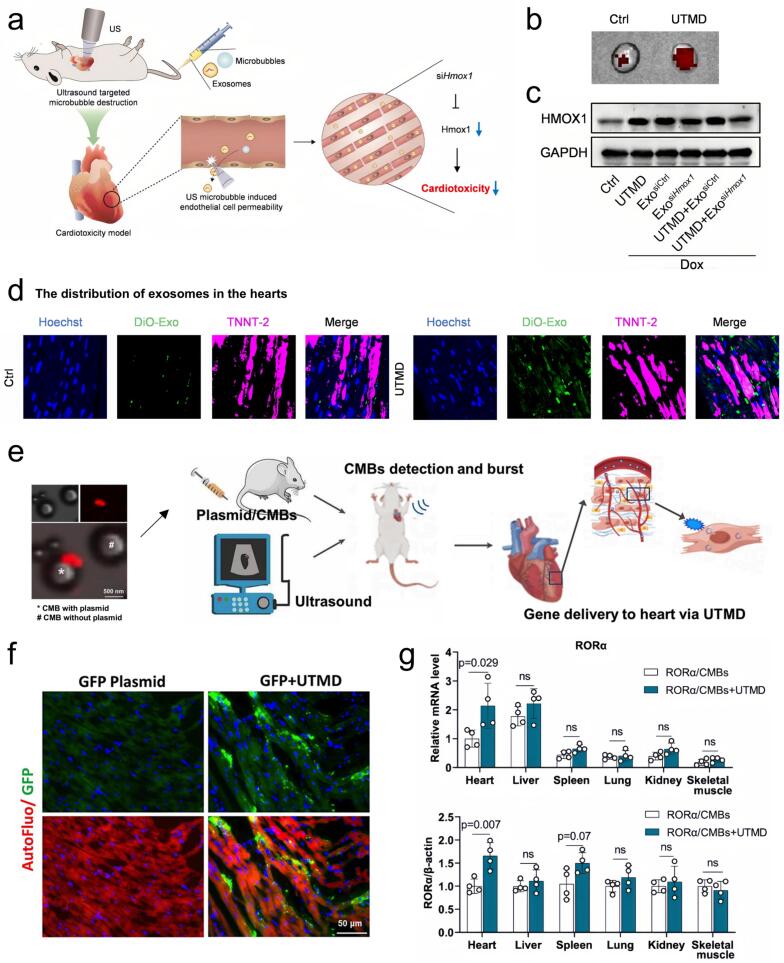
a) Schematic illustrating utmd-assisted exosome-mediated sihmox1delivery, inhibiting doxorubicin (DOX)-induced iron-dependent apoptosis in cardiomyocytes. b) Fluorescence images showing exosome localization in hearts of control versus UTMD-treated mice. c) The knockdown efficiency of siHmox1 in vivo in different treated mice as measured by qPCR. d) Representative images of exosomes localization in TNNT-2 positive cardiomyocytes [176]. Copyright 2024, Springer Nature. e) Illustration of UTMD-mediated RORα transport to the myocardium. f) UTMD-mediated GFP plasmid transfection visualized by fluorescence microscopy; nuclei stained with DAPI (blue) and GFP signal in green. g) Relative RORα mRNA expression in the cardiac and extra-cardiac tissues [177]. Copyright 2023, Springer Nature.

The development of MBs has facilitated enhanced targeting and delivery of therapeutic agents at both vascular and cellular levels. Excitation of MBs by ultrasound increases vascular and cellular permeability, allowing drugs and genes to cross the endothelial barrier and reach the cytoplasm for transfection [[155], [156], [157]]. The precise spatial and temporal control of ultrasound energy, in conjunction with MBs, enables targeted delivery of therapeutic agents to specific sites, thereby reducing off-target effects and minimizing systemic toxicity. [26,158]. The UTMD approach offers a non-invasive strategy for delivering bioactive agents directly to targeted tissues with high precision. The technique leverages low-frequency ultrasound to trigger in vivo MB cavitation, causing repeated expansion and contraction followed by bubble disruption. UTMD integrates diagnostic and therapeutic functions and is characterized by its precision, high efficiency, safety, and good repeatability [[159], [160], [161], [162]]. The mechanical effects generated by UTMD, including shear stress, microjets, and shock waves, induce cavitation and transient membrane poration, enabling enhanced cellular uptake [163,164]. By inducing transient pore formation and triggering endocytosis, UTMD facilitates efficient intracellular delivery of bioactive agents. The contribution of endocytosis is size-dependent, making UTMD a promising carrier for site-specific delivery of drugs and genes [165]. Additionally, UTMD enables transient opening of the blood–brain barrier (BBB) and blood-tumor barrier, permitting immunotherapeutic agents to access and accumulate at tumor sites. This capability reduces off-target toxicity in tumor immunotherapy [166,167]. A growing body of research has demonstrated the utility of UTMD in improving the efficiency of drug targeting and localized delivery to specific tissues [168,169]. For example, by injecting siHMOx1-coated exosomes into the cardiac region, UTMD enables targeted delivery of siHMOx1 to block doxorubicin (DOX)-induced iron-mediated cell apoptosis and cardiotoxicity (Fig. 6 a–d). Further studies have demonstrated that cardio-targeted delivery of the nuclear receptor RORα via UTMD optimizes the treatment of sepsis-induced cardiomyopathy with conventional doses of melatonin. UTMD-facilitated in vivo delivery of plasmid/cationic microbubble (CMB) resulted in detectable GFP in the heart three days post-treatment, correlating with marked upregulation of RORα at both the transcript and protein levels (Fig. 6 e–g). Studies have shown that combining UTMD with targeted, drug-encapsulated nanoparticles greatly increases cellular uptake under in vitro conditions and extends drug persistence in vivo. In a mouse pancreatic tumor xenograft model, the combination of PTX-NP-anti-CA19-9 NPs with UTMD produced the greatest tumor growth inhibition, while also optimizing pharmacokinetic behavior by extending the mean residence time (MRT) and decreasing systemic clearance [170]. Cationic microbubbles have been utilized as effective carriers to enhance the delivery efficiency and specificity of targeted genes to ischemic myocardium, enhancing DNA carrying capacity and improving gene transfection for cardiac regeneration. UTMD-mediated cationic microbubble (CMB) gene delivery significantly enhanced transfection efficiency and gene expression compared to commercial Definity microbubble technology [171]. In another study, focused ultrasound significantly improved the systemic administration and biological distribution of oncolytic viruses (OVs) co-injected with microbubbles via inertial cavitation. By combining efficient gene transfer with imaging capability, this approach produced a fiftyfold rise in tumor transgene expression, maintained tissue integrity, and facilitated real-time tracking of viral vectors [172].

UTMD treatment has also demonstrated potential in reducing immunotherapy-related adverse events. The technique has demonstrated the capacity to modulate the tumor microenvironment (TME) and improve cancer immunotherapy outcomes [163,173]. The integration of ultrasound with tumor-targeted MBs induces the formation of transient membrane pores, facilitating controlled gene transfer and defined local transfection. This approach efficiently transfects tumor and stromal populations, promoting cytokine-mediated immune activation, enhancing T-cell recruitment, and reducing tumor mass [70]. In another example, hepatic delivery of plasmid DNA via ultrasound-assisted MBs has shown significant therapeutic efficacy in murine models of ornithine transcarbamylase (OTC) deficiency. Ultrasound-enhanced plasmid transfer into fetal mouse liver corrected defects in ammonia metabolism, highlighting its potential as a temporary therapeutic strategy for OTC-deficient infants prior to liver transplantation [174]. Additionally, PD-L1 Ab/miR-34a-MBs have demonstrated the ability to deliver miR-34a to cervical cancer, inducing apoptosis through suppression of the anti-apoptotic protein Bcl-2 and activation of the pro-apoptotic protein Bax. This combination therapy has been shown to enhance T lymphocyte proliferation and promote CD8 + T cell infiltration into the tumor microenvironment, therby potentiating the overall antitumor immune response. The combination of anti-PD-L1 antibody-conjuncted MBs and ultrasound-mediated delivery of miR-34a represents a promising approach for promoting tumor apoptosis and enhancing immune regulation [175].

## 5 Biomedical applications of ultrasound-enhanced gene transfection

### 5.1 Tumors

Cancer continues to represent one of the foremost public challenges worldwide in the 21st century, presenting major social, public health, and economic burdens. As of 2022, global statistics indicated nearly 20 million individuals are newly diagnosed with cancer each year, resulting in approximately 10 million deaths [178]. Characterized by its high heterogeneity and complexity, cancer continues to be the primary cause of death across the globe. The management of cancer involves intricate and multi-dimensional therapeutic strategies, particularly due to the tumor microenvironment (TME), which consists of a heterogeneous network of stromal cells. Paracrine signaling between cancer cells and stromal components often lead to a TME that favors tumor growth, requiring the development of more sophisticated treatment strategies setting a continues race that contribute to negative patient outcomes as reflected by the increased mortality rate [179,180]. In contrast to the regular vascular systems found in normal tissues, blood vessels within tumors are frequently abnormal, exhibiting features such as dilated capillaries, leaky walls, and slow blood flow. Tumor growth is further compounded by the need for continuous angiogenesis to support new blood vessel formation, making the TME hypoxic in nature [181]. Hypoxia is a central process in tumor biology, with the hypoxia-inducible factor (HIF) transcription factors enabling tumor cells to adapt to low-oxygen conditions. Moreover, hypoxia enhances PD-L1 expression, contributing to an immunosuppressive environment [182]. Tumor heterogeneity, driven by genetic mutations, environmental factors, and irreversible cellular changes, often complicates the treatment process and contributes to therapeutic resistance [183]. Surgery, chemotherapy, and radiotherapy remained as the main traditional cancer therapies and first line of defense against cancer; however, these approaches often lead to significant side effects and fail to completely eradicate the tumor, especially due to the presence of the TME, which can hinder treatment efficacy. More recent advances, such as stem cell-based interventions, precised-targeted therapies, thermal or chemical ablation, nanomedicine platforms, antioxidants compounds, and sonodynamic therapy, have shown potential in overcoming some of these limitations [[184], [185], [186]].

**Fig. 7:**
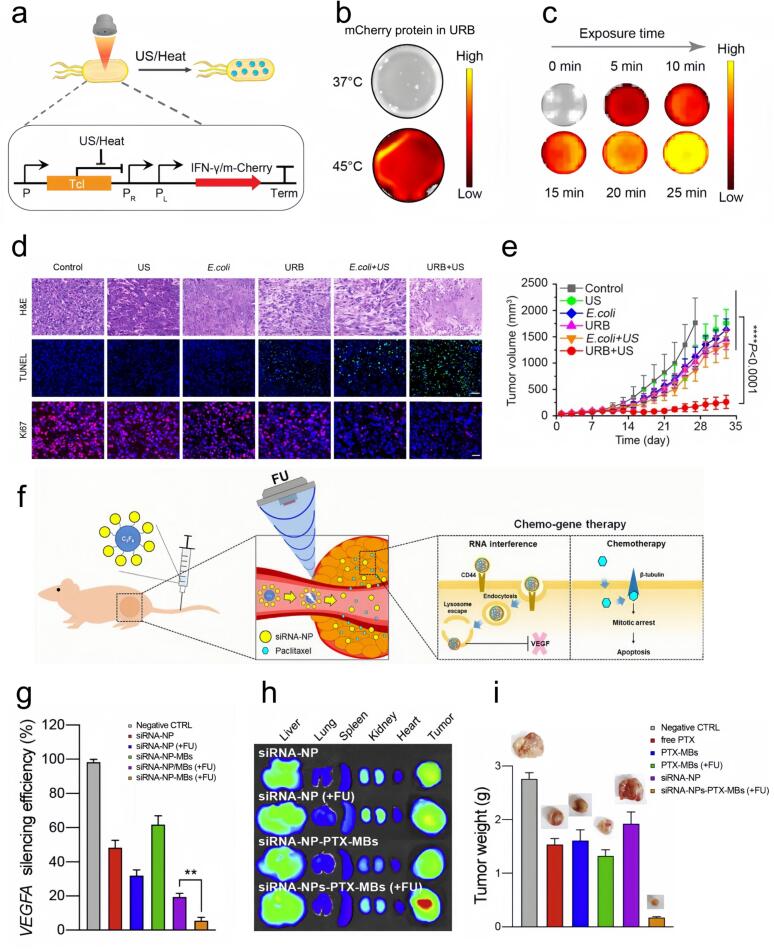
a) Schematic representation of mCherry/cytokine interferon-γ (IFN-γ) co-expression mechanism based on the pBV 220 plasmid in bacteria containing a focus ultrasound-induced temperature initiation therapy loop. b) Temperature-dependent Fluorescence of mCherry protein in URB. c) Time-dependent mCherry fluorescence in URB maintained at 45 °C. d) H&E, TUNEL and Ki 67 staining of tumor sections under different treatment conditions. e) Tumor growth curves of mice receiving different treatments [208]. Copyright 2022, Springer Nature. f) Illustration of the SiRNA-NP and drug-carrying microbubbles nanocomplex triggering enhanced efficacy of chemical gene therapy under focused ultrasound (FU). g) Relative vascular endothelial growth factor (VEGF) mRNA expression in B16F10 cell in different treatment groups. h) Organ-specific accumulation of siRNA-NPs assessed ex vivo. i) Tumor burden assessed by weight of excised tumor tissues among groups [209]. Copyright 2020, Elsevier B.V.

Ultrasound-based therapies offer new opportunities to manipulate the TME and enhance cancer treatment. The thermal and mechanical effects of ultrasound, including hyperthermia, ablation, histotripsy, and microbubble cavitation, can modulate the TME, stimulate antitumor immune response, and suppress tumor progression. By transiently disrupting vascular barriers, ultrasound-induced microbubble cavitation facilitates the infiltration of immune effector cells, cytokines, tumor-associated antigens, monoclonal antibodies, and therapeutic nucleic acids into to tumor tissues. Additionally, intense cavitation can induce direct tumor cell disruption, leading to the release of intracellular antigens and promoting the activation of antigen-presenting cells, which in turn triggers adaptive immune responses [[187], [188], [189], [190], [191], [192], [193]]. Focused ultrasound can also modulate critical features of tumor biology, including alleviating hypoxia, increasing vascular permeability, and reducing interstitial fluid pressure (IFP), thereby improving therapeutic agents distribution within tumors and improving treatment outcomes [ [194]. Therapies combining ablation with immune stimulation, such as high-intensity focused ultrasound (HIFU), hold significant promise in boosting immune responses to cancer. HIFU induces tumor tissue necrosis, releasing tumor-associated antigens and DAMPs which trigger antigen presentation and subsequent activation of adaptive immunity. The use of HIFU in combination with immune modulation may lead to systemic, long-lasting anti-tumor immunity [[195], [196], [197], [198]]. Evidence indicates that low-intensity focused ultrasound (LIFU) mitigates inflammatory processes while stimulating cellular repair mechanisms in multiple pathological contexts. LIFU promotes anti-inflammatory activity through transcriptional activation of immune-suppressive genes, including those involved in regulatory T cells (Tregs), mesenchymal stem cells (MSCs), and myeloid-derived suppressor cells (MDSCs) [199]. Furthermore, ultrasound offers a means to control gene expression dynamics in bacteria, which is advantageous for developing ultrasound-responsive bacteria (URBs) for cancer therapy. For instance, brief exposure to focused ultrasound-mediated hyperthermia stimulates IFN-γ gene transcription, which in turn potentiates the therapeutic effects of URBs across preclinical models(Fig. 7 a–e). Ultrasound has also been shown to upregulate microRNA-124 expression, which inhibits STAT3 activation, leading to reduced migration and metastasis in colon cancer models [200]. A promising approach involves the use of microbubbles (MBs) dual-loaded with antigen mRNA and immunomodulatory TriMix mRNA for ultrasound-triggered dendritic cell (DC) transfection. This technique triggers targeted T-cell activation against tumor antigens and significantly limiting tumor expansion while promoting long-term antigen-specific immune memory. In vivo studies have shown that approximately 30 % of vaccinated animals experienced complete tumor elimination and sustainable immune protection, suggesting the potential of ultrasound-enhanced DC vaccines as an effective cancer immunotherapy [201]. Additionally, IL-12 gene therapy, when combined with bubble liposomes and ultrasound, has shown efficacy in eliciting robust antitumor immune responses. The use of ultrasound to deliver IL-12 plasmids significantly enhanced CD8(+) T-cell migration and inhibited tumor growth in mouse models [202]. A novel nanocomplex combining siRNA-containing nanoparticles (siRNA-NPs) and chemotherapeutic-loaded MBs, responsive to focused ultrasound (FU), has demonstrated enhanced therapeutic efficacy. Upon ultrasound exposure, the nanocomplexes collapse around the tumor site, allowing siRNA and chemotherapeutics to penetrate the dense extracellular matrix (ECM) and enhance both gene and chemotherapy outcomes (Fig. 7 f–i). Ultrasound-assisted MB delivery of miRNA-122 and anti-miR-21 alongside doxorubicin, has shown promising results in inhibiting hepatocellular carcinoma (HCC) tumor growth, while reducing the necessary dose of chemotherapy drugs [203]. The blood–brain barrier (BBB) continues to pose a major obstacle in the effective delivery of therapeutic agents for brain tumors such as glioma. However, transcranial MBs-enhanced FUS (MB-FUS) has demonstrated its ability to safely and controllably open the BBB, enhancing the delivery and bioavailability of therapeutic agents within brain tumors. In patients with invasive glioma, MR-guided FUS (MRgFUS) was shown to enhance drug delivery across the BBB, allowing effective treatment and tumor growth inhibition [204]. Furthermore, MB-FUS has been used in combination with cationic lipid-polymer hybrid nanoparticles (LPHs) to facilitate the efficient delivery and cellular uptake of siRNA within brain tumor tissue, significantly increasing tumor cell death and offering unique therapeutic advantages for brain tumor treatment [205,206]. In glioblastoma multiforme (GBM) therapy, MB-FUS-mediated delivery of gene-supported lipid-polymer hybrid nanoparticles (LPHNs) has demonstrated the ability to potentiate the antitumor efficacy of temozolomide (TMZ), reduce tumor growth, and prolong survival in experimental models. By promoting localized nanoparticle delivery, this method augments therapeutic efficacy and simultaneously minimizes systemic toxicity, enhancing overall biosafety [207].

### 5.2 Cardiac insufficiency

Cardiac insufficiency is caused by various factors that lead to a decline in myocardial systolic function, which reduces forward blood output, resulting in circulatory stagnation in both systemic and pulmonary vascular beds and subsequently manifesting as related symptoms [210]. As cardiac insufficiency progresses, it can lead to heart failure (HF), a primary cause of mortality in developed countries, with the global prevalence of patients continuing to rise. Coronary heart disease is often a primary contributor to cardiac insufficiency, especially when it causes myocardial infarction. The localized necrosis of the myocardium due to infarction may lead to the loss of the cardiac pumping function, which subsequently results in cardiac insufficiency, remaining a leading cause of death globally [211]. Acute coronary syndromes (ACS) are most commonly attributed to thrombosis in coronary artery disease, typically associated with the development of lipid-laden atherosclerotic plaques in the coronary arteries, putting patients at risk for unstable angina, acute myocardial infarction (MI), and cardiogenic death [212]. Ultrasound serves a key function in cardiovascular medicine, acting not only as a diagnostic tool for cardiovascular diseases but also for prognostic assessment. Intravascular angiographic MBs are visualized via acoustic cavitation, which generates detectable changes in reflected ultrasound. Under high-intensity conditions, acoustic oscillations produce sufficient shear forces to enhance endothelium-mediated perfusion in thrombotic and atherosclerotic vessels. Ultrasound-targeted MB cavitation can effectively resolve microvascular obstruction, activate endothelial nitric oxide synthase (eNOS), and increase endothelial nitric oxide (NO) bioavailability. The oscillations and ruptures of MBs in response to ultrasonic waves can also induce microstreaming and jetting effects, which help to disrupt thrombi [54,213,214]. A novel sonothrombolysis method, which treats emboli without the use of thrombolytic drugs, offers an effective treatment for thrombotic and embolic by efficient entrapment and disruption of blood clots [215]. Clinical evaluation of catheter-mediated ultrasound therapy has demonstrated its effectiveness and safety. The cumulative duration of pulsed wave (PW) ultrasound treatment directly influences the efficiency of thrombolysis. Under optimal PW parameters and intensity, thrombolytic efficiency can reach 91 %, with complete thrombolysis and intact arterial walls after 2 min of treatment [216]. During intravenous MB infusion (ultrasonic thrombolysis), cavitation of MBs caused by high mechanical index (MI) pulses delivered via diagnostic transducers creates shear forces that dissolve coronary artery and microvascular thrombi, thus re-establishing both epicardial and microvascular perfusion in patients with acute ST-segment elevation myocardial infarction (STEMI) [[217], [218], [219]]. Ultrasound thrombolysis in STEMI patients has demonstrated that the addition of ultrasound thrombolysis to percutaneous coronary intervention (PCI) can increase recanalization rates, lead to smaller infarcted areas and improve post-STEMI systolic performance [220]. Moreover, ultrasonic thrombolysis can reduce microvascular obstruction and improve myocardial dynamics in STEMI patients [221].

**Fig. 8:**
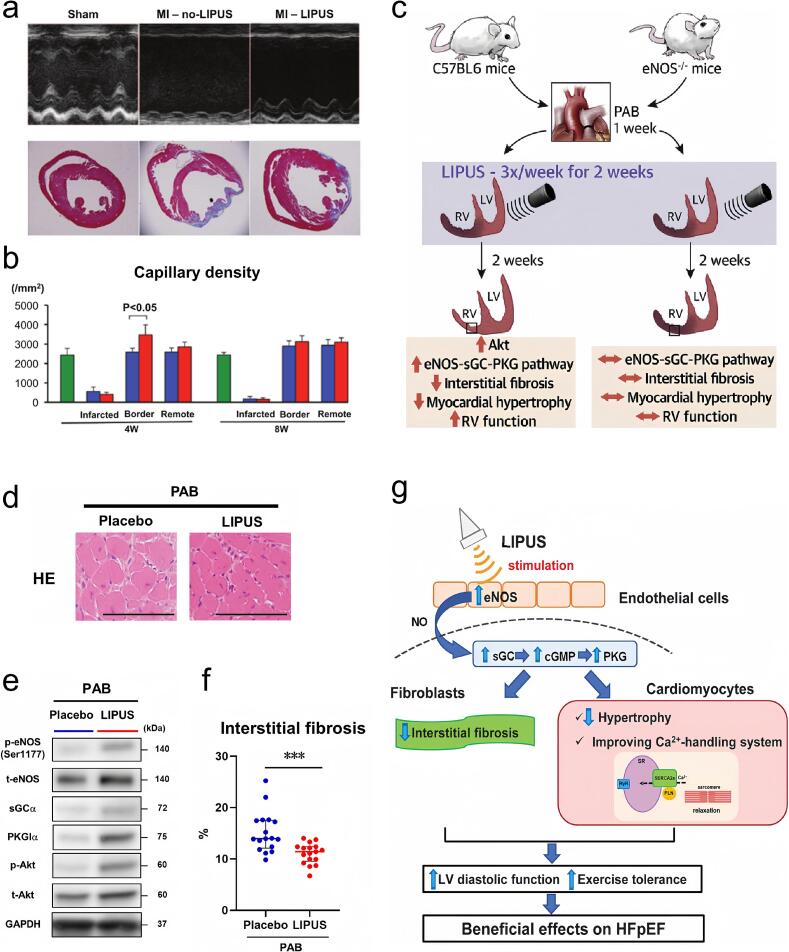
a) The upper shows the m−mode echocardiogram 8 weeks after acute myocardial infarction. The lower presents the Masson staining of cardiac sections, which displays the areas of infarction in different groups. b) Diagram showing the density of capillaries in different groups [229]. Copyright 2016, Wolters Kluwer Health. c) Illustration of LIPUS treatment improved RV dysfunction in preclinical models associated with endothelial nitric oxide synthase (eNOS) activation. d) Representative image of cardiac tissue following HE staining. e) Representative Western blot illustrating the levels of eNOS, phosphorylated eNOS at Ser1177, sGCα, PKG Iα, Akt, and p-Akt in whole RV from mice. f) Quantitatively analyzed the area of myocardial fibrosis [230]. Copyright 2023, Elsevier. g) Schematic of LIPUS-mediated improvement of cardiac diastolic dysfunction in mice through the eNOS-NO-cGMP-PKG pathway and the cardiomyocyte Ca^2+^ regulation [231]. Copyright 2020, Oxford University Press.

Furthermore, the prognosis of coronary heart disease, particularly post-infarction left ventricular remodeling remains a critical issue in cardiovascular care. LIPUS has been shown to promote early post-infarction activation of VEGF, eNOS, pERK, and pAkt signaling pathways in the affected myocardial tissue, promoting angiogenesis and improving left ventricular function in infarcted mice (Fig. 8 a–b). Studies have also indicated that low-energy extracorporeal shockwave (SW) therapy significantly enhances VEGF expression, improving myocardial angiogenesis and alleviating ischemia in chronic myocardial ischemia models and clinical cases of severe angina [222,223]. SW treatment has been shown to ameliorate symptoms, enhance exercise tolerance, and restore myocardial perfusion in severe coronary artery disease patients, while maintaining a favorable safety profile. Right ventricular failure (RVF) is a clinical condition primarily resulting from elevated right ventricular (RV) afterload, exhibited through a decreased right ventricular ejection fraction (RVEF) and RV dilation. During prolonged in vivo hypoxia, RV function in eNOS-deficient (eNOS-/-) mice deteriorates significantly. Downregulation of endothelial nitric oxide synthase (eNOS) is observed in individuals suffering from RVF. Low-intensity pulsed ultrasound (LIPUS) effectively improves eNOS expression and its downstream pathway via eNOS-mediated pathways, making it a promising therapeutic target for RVF. LIPUS therapy has demonstrated significant improvements within pulmonary arterial banding (PAB) −induced RV dysfunction in mice, notably improving cardiac hypertrophy and interstitial fibrosis (Fig. 8 c–f). The incidence of heart failure characterized by preserved left ventricular ejection fraction (HFpEF) has risen dramatically and represents a major public health concern globally [[224], [225], [226]]. Diastolic dysfunction represents a central pathophysiological mechanism underlying in HFpEF, with its severity correlating with symptomatic heart failure and an increased risk of mortality [227,228]. LIPUS has been shown to promote angiogenic and reduce inflammation through its mechanically induced pathways, positioning it as a promising non-invasive intervention for cardiovascular diseases. LIPUS therapy has been shown to improve diastolic dysfunction in mice by enhancing the eNOS-NO-cGMP-PKG signaling cascade and Ca^2+^ homeostasis in cardiomyocyte. LIPUS treatment in 12-week-old obese diabetic mice (db/db) preserved systolic function, significantly improved cardiac diastolic function, improved tissue-level relaxation characteristics and motor capabilities, and attenuated cardiomyocyte enlargement and interstitial fibrotic remodeling (Fig. 8g).

### 5.3 Stroke

**Fig. 9:**
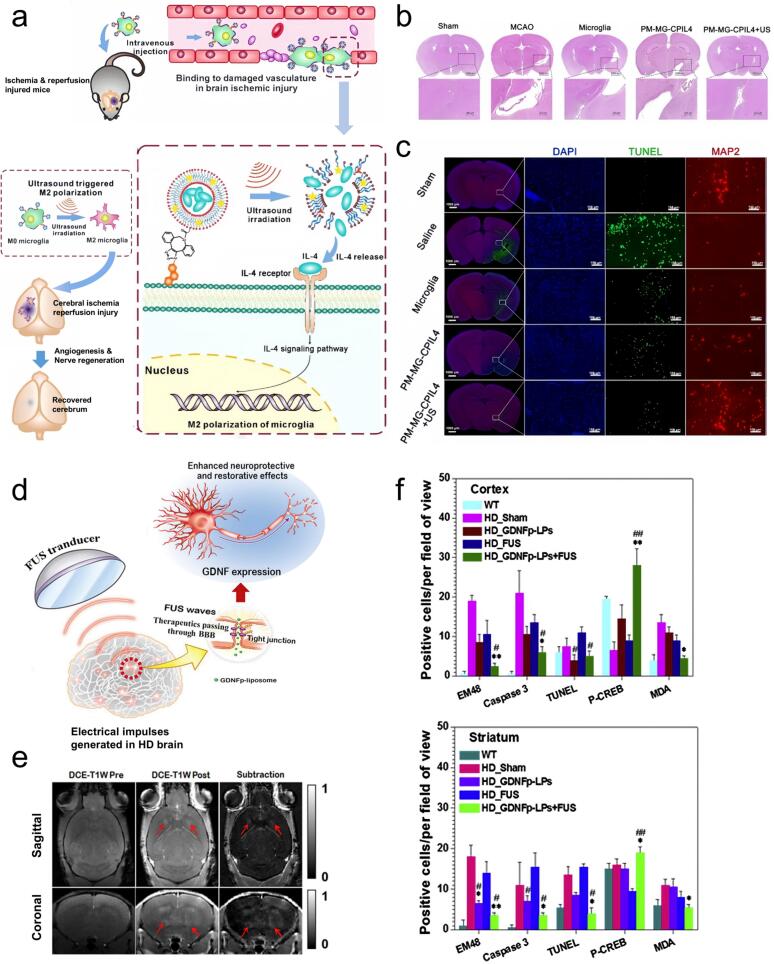
a) Schematic illustration of ultrasound-mediated platelet-microglial fusion for targeted polarization therapy in ischemic reperfusion injury after stroke. b) H&E-based pathological assessment of brain tissue from middle cerebral artery occlusion (MCAO) mice. c) Immunohistochemical analysis was used to evaluate neuronal degeneration and apoptosis in ischemic brains after differen treatments. Fluorescence images of neurons (MAP2) and apoptotic cells (TUNEL) with DAPI in the stroke site [239]. Copyright 2020, Wiley‐VCH GmbH. d) Conceptual schematic of focused ultrasound (FUS) −assisted delivery of glial cell line-derived neurotrophic factor plasmid liposomes (GDNFβ-LPs) in HD mouse models. e) Representative MR images of 6-week-old R6/2 HD mice pre- and post-FUS BBB disruption. f) Evaluation of protein aggregates, Caspase-3 activity, TUNEL staining, P-CREB and MDA in the tissues of mice in different treatment groups [240]. Copyright 2019, Elsevier Inc.

Stroke, a neurological disorder with a poor clinical prognosis, characterized by a pronounced inflammation and immune activation following the event. This inflammatory cascade often leads to severe sequelae and high mortality rates [232,233]. For ischemic stroke, the current gold standard treatment is the prompt intravenous administration of tissue plasminogen activator (tPA), which dissolves the thrombus and restore cerebral perfusion. Ultrasound serves an important function in facilitating thrombolysis, strengthening the influence of tPA by breaking down fibrin, increasing enzyme transport, dilating arteries, and improving the absorption and penetration of tPA into the thrombus. Sonothrombolysis, a novel and safe therapeutic approach, has shown potential for acute stroke, with microspheres providing evidence for enhanced ultrasound-mediated thrombolysis [234]. The latest generation of microspheres (mS) contains specific phospholipids that can be detected and activated by ultrasound. Upon intravenous injection, these microspheres approach and penetrate the thrombus. The liquid jets produced by ultrasound-induced cavitation erode the thrombus surface, increasing its exposed area. In the presence of tPA, this mechanical erosion accelerates clot dissolution. Microsphere-enhanced ultrasound thrombolysis is capable of breaking down the thrombus into small micron-sized particles without causing embolism or re-blockage of blood vessels [235,236]. Ultrasound at lower frequencies exhibits enhanced transcranial penetration, and animal studies have demonstrated its ability to accelerate tPA-mediated thrombolysis in the brain's vascular areas. However, the biological effects of low-frequency ultrasound may increase the risk of cerebral hemorrhage in patients receiving intravenous tPA [237]. Sonothrombolysis using recombinant tissue plasminogen activators (rtPA) and microbubbles (MBs) has undergone extensive evaluation regarding its ability to augment thrombolytic therapy. Nanoparticles with various shapes exhibit differing reactivities to cavitation, leading to varied thrombolytic potentials. For example, cavitation-assisted sonothrombolysis using asymmetric gold nanostars (NSt) has shown promise in treating acute ischemic stroke by enhancing acoustic stimulation and microbubble-mediated cavitation. The co-administration of NSt and MBs significantly reduced infarct size in a thrombus model and was beneficial for the recovery of cerebral blood flow [238]. Furthermore, when exposed to ultrasound, the combination of platelets and microglia can be directed toward an anti-inflammatory state, thereby facilitating localized neural repair after stroke. The artificially designed microglia with human platelet membranes demonstrates high affinity for injured cerebral vasculature and enables controllable anti-inflammatory polarization through ultrasound-triggered IL-4-encapsulated liposomes. This approach contributes to sustained rehabilitation by limiting neuronal apoptosis, enhancing the generation of new neurons, and improving functional restoration [239]. A study evaluating the therapeutic efficacy of PM-MG-CPIL4 (comprising platelet membrane decorated M0 microglia with IL-4 shielding in liposome carrying the sonosensitizer protoporphyrin IX) in ischemic stroke/reperfusion (I/R) models demonstrated promising results. In murine models of middle cerebral artery occlusion (MCAO), systemic administration of PM-MG-CPIL4 exhibited enhanced accumulation within the damaged brain. In the PM-MG-CPIL4 + US treatment group, survival rates were significantly improved, infarct size reduced, and neurons positive for microtubule-associated protein 2 (MAP2) in the ischemic brain showed the greatest increase. Exposure to ultrasound enabled PM-MG-CPIL4 to deliver significant protective effect on brain cells post-stroke, facilitating recovery (Fig. 9 a–c).

### 5.4 Neurodegenerative diseases

As with cardiovascular diseases and cancer, the prevalence of neurological disorders is rising in aging population. For patients with neurological conditions, ultrasound ablation of tumors offers advantages over traditional methods such as surgical resection and radiation therapy, as it is non-invasive and causes minimal damage to surrounding healthy brain tissue. For patients with essential tremor or Parkinson's disease, beyond deep brain stimulation (DBS) and conventional thalamotomy, the development of magnetic resonance imaging-guided FUS (MRgFUS) has become a promising frontier for treating deep brain lesions [241]. The blood–brain barrier (BBB) poses a significant challenge in neurological disorder therapy by restricting the transport of drugs to brain tissue [242]. However, reversible opening of the BBB via MRI-guided low-intensity focused ultrasound (LIFU) has been reported in multiple neurological conditions, such as Alzheimer's disease (AD) [243], Parkinson's disease [244], brain tumors [204,245], and amyotrophic lateral sclerosis [169], among others [246]. Serving as a key focus for therapeutic development, the hippocampus is critically implicated in AD, epilepsy, and depression. LIFU has been reported to mediate safe, noninvasive, and transient BBB opening repetitively within the human hippocampus and entorhinal cortex (EC) [[247], [248], [249]]. Reducing amyloid beta (Aβ) accumulation in the brain is a key therapeutic strategy in Alzheimer's disease. FUS allows transient modulation of BBB permeability, enhancing the removal of amyloid from specific brain regions. The reduction of Aβ levels in treated areas is significantly greater relative to corresponding areas in the opposite hemisphere, which remain untreated [250]. MR-guided FUS (MRgFUS), in combination with intravenous microbubbles, serves to induce targeted, transient BBB opening in individuals with neurodegenerative disorders and brain tumors, such as Parkinson's disease. Following MRgFUS treatment in Parkinson's disease-related dementia (PDD) patients, mild cognitive improvements were observed, showing no significant differences on amyloid or fluorodeoxyglucose (FDG) PET scans. Thus, BBB disruption induced by MRgFUS in PDD has been shown to be safe, transient, and reproducible [244].

Huntington's disease (HD) is a neurodegenerative disorder inherited in an autosomal dominant manner that has also been targeted for novel therapeutic strategies. A gene-liposomal system was developed in which DPPC liposomes (LPs) carried glial cell line-derived neurotrophic factor (GDNF) plasmid DNA (GDNFβ). This GDNFβ-liposome (GDNFβ-LPs) complex was tested in transgenic mouse models of HD, both before and after symptom onset. Pulsed focused ultrasound (FUS) exposure was utilized to transiently disrupt the BBB, enabling targeted, noninvasive, and non-viral gene delivery into the central nervous system (CNS) for therapeutic applications. FUS-facilitated GDNFβ-LPs therapy led to substantial improvements in motor function in HD mice. Overexpression of GDNF led to a substantial reduction in polyglutamine-amplified aggregates, mitigated oxidative damage and cell death, promoted neurite extension, and supported neuronal viability (Fig. 9 d–f). This approach demonstrates that GDNFβ-LPs + FUS can achieve therapeutic gene delivery by supporting localized exosmosis and targeted distribution, offering a potential non-invasive treatment for central nervous system diseases [240].

### 5.5 Musculoskeletal disorders

**Fig. 10:**
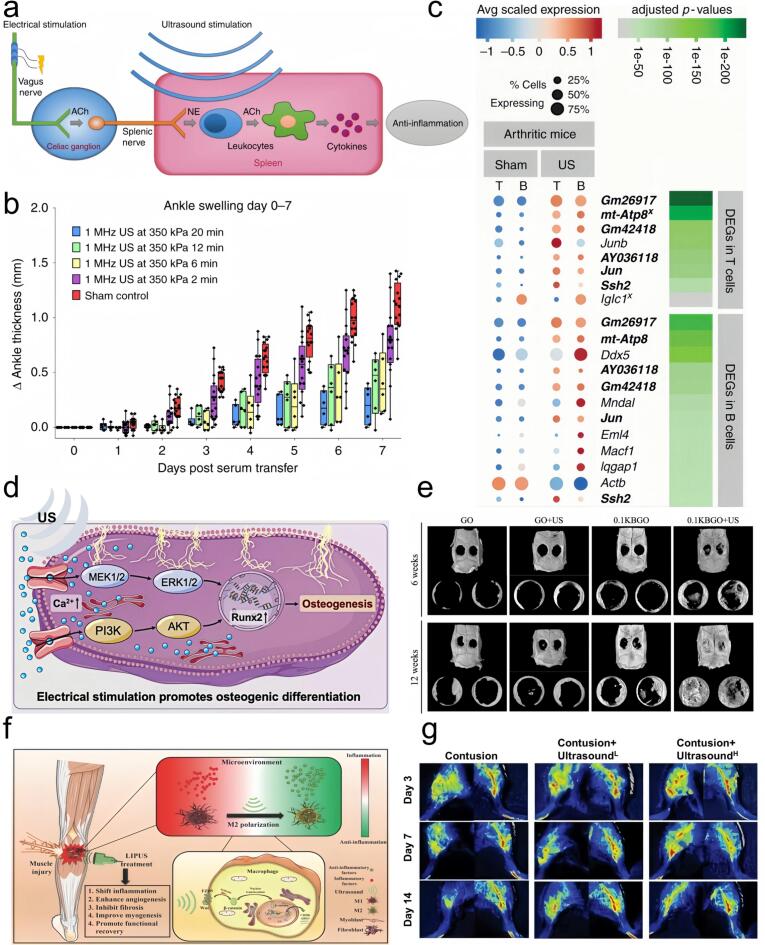
a) Modulation of the cholinergic anti-inflammatory pathway through vagal electrical or splenic ultrasonic stimulation promotes an anti-inflammatory state. b) The duration of ultrasound has a dose-dependent effect on arthritis. c) Spleen T and B cells showed genetic induction after US treatment [253]. Copyright 2019, Springer Nature. d) Schematic diagram of osteogenic differentiation promoted by electrical stimulation under ultrasonic stimulation. e) Cross-sectional bone regeneration in various groups was observed via micro-CT at 6 and 12 weeks after surgery [270]. Copyright 2024, Springer Nature. f) Diagram depicting pathophysiological and molecular mechanisms of LIPUS in the treatment of muscle injury. LIPUS reduces inflammation, enhances angiogenesis, inhibits fibrosis, and improves muscle production in the body. g) Bilateral representative perfusion images of gastrocnemius muscle. LIPUS causes more physiological blood perfusion on day 14 [280]. Copyright 2023, Ivyspring International Publisher.

Arthritis refers to a category of inflammatory disorders that primarily impact the joints and periarticular tissues, resulting from diverse etiological factors including but not limited to inflammatory processes, infections, degeneration changes, traumatic injuries, or other pathological conditions. Autoimmune diseases, for example, rheumatoid arthritis, can be improved by stimulating cholinergic anti-inflammatory pathways [251,252]. Daily targeted ultrasound stimulation of the spleen at specific intensities has been shown to significantly reduce disease severity in mice with inflammatory arthritis. Ultrasound stimulation of the spleen offers protective and therapeutic effects, suggesting its potential for treating inflammatory diseases. The therapeutic benefits of ultrasound are believed to depend on lymphocytes, with spleen T and B cells contributing to anti-inflammatory responses, either through vagus nerve electrical stimulation or direct ultrasound stimulation of the spleen. The spleen stimulation of ultrasound can induce notable changes in lymphocyte transcription profiles, leading to differential gene expression [253] (Fig. 10 a–c). Osteoarthritis, despite its inability to achieve a cure, often requires non-surgical treatments such as analgesics and anti-inflammatory drugs to relieve symptoms, though it does not cure the disease [254]. According to a study, an injectable and biodegradable piezoelectric hydrogel, composed of short electrospun poly-L-lactic acid nanofibers integrated within a collagen matrix, produces localized electrical signals upon articular injection and ultrasound activation. Cartilage formation had been promoted by stimulation which induced stem cell secretion of TGF-β1and enhanced cell migration. This approach provides a novel direction for the treatment of osteoarthritis [255].

Patients with bone defects caused by trauma, tumors, or specific pathological conditions like chronic inflammation and necrosis present significant physical and mental burdens [256,257]. The goal of current bone tissue engineering methods is to reduce procedural trauma, stabilize affected bone, and create a favorable osteogenic microenvironment [258]. Autogenous bone grafting (ABG) is still regarded as the gold standard in bone regeneration. However, addressing critical-size bone defects with complex geometries is still a key difficulty in orthopedic practice [259]. To address this issue, implants with robust osteogenic properties and complex defect adhesion capabilities are essential, such as programmed RNA nanomachines [260], dopamine-deposited fuzzy microspheres [261], and hydrogels [262]. As a form of biophysical stimulation, endogenous bioelectrical cues in the bone microenvironment contribute significantly to the regeneration of bone defects [263]. Pulsed electric fields, when applied at appropriate intensity and frequency, can promote osteogenic gene activation and support tissue cell growth and differentiation in defects, and facilitate bone regeneration. Electric bone growth stimulation (EBGS) can serve as a targeted strategy to accelerate bone regeneration and support successful fusion [264]. The advancement of bioactive materials for orthopedic implants aimed at improving bone repair has been a major research priority. The ideal implant material should exhibit bioactivity similar to natural bone [265]. Owing to their outstanding biocompatibility, resistance to corrosion, and favorable mechanical characteristics, titanium and its alloys are extensively employed in both dental and orthopedic implants [266]. Bone formation and the healing of fractures are profoundly influenced by electrical signals [267]. Barium titanate (BT), a highly biocompatible electroactive material, generates electrical signals when subjected to mechanical forces, without causing inflammatory responses or foreign body responses at the site of implantation [268,269]. A composite designated BT/TC4 is formed through the deposition of BT piezoelectric ceramic onto the surface of TC4 titanium alloy, was studied under LIPUS stimulation. Electrochemical measurements showed that BT/TC4 when stimulated by LIPUS, produced a microcurrent of around 10 μA/cm^2^. In vitro studies revealed that LIPUS enhanced osteogenesis in MC3T3-E1 cells, supporting cell adhesion, expension, and phenotypic maturation, and increasing intracellular calcium concentrations through the activation of L-type calcium channels. Consequently, this piezoelectric BT/TC4 material, in combination with LIPUS, promotes osteogenesis and has potential as a treatment for early bone-implant contact formation [55]. However, the brittleness and low flexibility of piezoelectric ceramics limit their application in irregular fracture sites. To overcome this, a nanocomposite hydrogel has been synthesized through dynamic covalent bonds formed between amine-modified piezoelectric nanoparticles and biopolymer hydrogel matrices. This injectable hydrogel is shape-adaptive, highly adherent to bone, and actuated by ultrasound, providing a promising solution for treating irregular bone defects. Ultrasound-triggered nanocomposite hydrogels produce controlled electrical outputs (−41.16 to 61.82 mV), markedly promoting bone-forming activities under both laboratory and living system conditions. The accelerated healing of critical-size skull defects in rats has been demonstrated. Additionally, ultrasound-responsive hydrogels increase intracellular calcium entry and upregulate PI3K/AKT as well as MEK/ERK signaling pathways, promoting osteogenic differentiation of bone mesenchymal stem cells (Fig. 10 d-e) [270]. The combination of localized ultrasound and microbubble-facilitated gene transfer to endogenous stem cells exhibits potential for enhancing fracture repair and bone regeneration. Ultrasound-mediated bone morphogenetic protein-6 (BMP-6) gene transfer achieved complete radiographic and functional recovery of fractures in every treated animal by six weeks after treatment, in contrast to nonunion in controls. Collectively, the data demonstrate that delivering genes to mesenchymal progenitor cells via ultrasound can successfully address nonunion in large animal models [271].

Traumatic injuries to skeletal muscle can compromise functional mobility, limit routine activities, and negatively affect quality of life. Muscle injuries present ongoing challenges in clinical reconstruction and regeneration [272,273]. Functional recovery depends critically on the formation and proper maturation of regenerated muscle fibers, and relies heavily on the activation and proliferation of satellite cells or myogenic progenitor cells (MPCs) [274]. Following acute muscle injury, pro-inflammatory M1 macrophages are first recruited to the lesion, where they clear necrotic tissue through phagocytosis and initiate myogenesis via the production of nitric oxide (NO) and pro-inflammatory cytokines [275]. The transition to M2 macrophages promotes muscle regeneration and differentiation after M1macrophages have completed their initial inflammatory role [[276], [277], [278]]. The equilibrium between M1 and M2 macrophage polarization is crucial for muscle healing [279]. LIPUS, at an intensity of 60 mW/cm^2^, has been shown to enhance skeletal muscle regeneration significantly by facilitating the shift of macrophages from M1 to M2. Furthermore, LIPUS can reduce inflammation, enhance angiogenesis, inhibit fibrosis, and improve muscle formation in vivo through multiple pathophysiological and molecular mechanisms (Fig. 10 g–f). Therefore, harnessing LIPUS to modulate macrophage polarization offers a promising therapeutic strategy for managing diverse muscle injuries and inflammatory conditions [280]. LIPUS treatment has shown to suppress pro-inflammatory cytokine production, restrict inflammatory cell infiltration, and regulate inflammatory cell phenotypes [281]. These effects may be associated with enhanced blood flow, activation of mitochondrial biogenesis, and antioxidant stress responses. Therefore, LIPUS can promote functional skeletal muscle repair by modulating the inflammatory microenvironment [282,283]. Studies have shown that LIPUS activates the FAK-ERK1/2 signaling pathway, stimulating bone marrow mesenchymal stem cell (BMSC) to migrate, expand, and differentiate, thereby accelerating tissue repair. Additionally, LIPUS treatment markedly increasess the expression of cyclin D1, E1, A2, and B1 via activation of the ERK1/2 and PI3K-Akt signaling pathways [[284], [285], [286], [287]].

## 6 Conclusion and perspectives

Continued advances in gene transfer technology have significantly influenced the development of modern molecular medical treatments. The versatility and effectiveness of multi-mode gene therapy have made it widely adopted and further developed in clinical practice. Gene therapy works by altering genes within cells and has become an option for diseases prophylaxis and therapy. The Food and Drug Administration (FDA) and the European Medicines Agency (EMA) evaluates the reliability and efficacy of gene therapy and are the regulatory bodies responsible for the review and authorization of gene therapy [288]. The efficacy and safety of gene vectors play a critical role in gene therapy, with long-term safety being one of the core concerns. Regulatory bodies now require long-term follow-up for participants in many gene therapy trials [289]. As clinical success stories continue to accumulate, ultrasound-mediated gene therapy approaches are expected to become increasingly significant. As an emerging therapeutic strategy, ultrasound-mediated gene therapy, during development of clinical transformation and application development, not only needs to overcome technical challenges but also urgently needs to systematically address the corresponding ethical and regulatory issues. This treatment approach has both potential therapeutic advantages and risks, especially showing broad clinical application prospects in the fields of malignant tumors and rare diseases [290]. In practical applications, ultrasound-mediated gene therapy is often combined with specific drugs (such as liposomes or nanoparticle carriers) to form a composite treatment system. In such combinations, ultrasound, as a non-biological component, is usually classified as a “medical device”, while gene therapy preparations are managed as biological products or drugs, resulting in the need for such combination products to follow differentiated regulatory pathways [291,292]. From the perspective of regulatory science, the combination of ultrasound with drug compounds or live cell therapy products significantly increases the complexity of the approval process. Such combination products are usually regarded as new therapeutic entities and need to undergo strict and comprehensive risk–benefit assessment. Their mechanism of action, safety and efficacy must be clearly defined to meet the review requirements of drug regulatory authorities for innovative combination therapies [293,294]. It is worth noting that the regulatory attribution of such ultrasound and drug combined products is primarily determined by their Primary Mode of Action (PMOA). For example, in accordance with the relevant regulations of the European Union, Drug-Device Combination products (drug-device Combination, DDC) are regulated respectively by (EU) 2017/745 (Medical Device Regulation) or 2001/83/EC (Medicines for Human Use Directive) based on their PMOA. Similarly, in compliance with the relevant regulations of the European Union, Drug-Device Combination products (drug-device Combination, DDC) are regulated respectively by (EU) 2017/745 (Medical Device Regulation) or 2001/83/EC (Medicines for Human Use Directive) based on their PMOA. As stated in Section 503(g) of the Federal Food, Drug, and Cosmetic Act, the FDA is required to allocate the primary regulatory authority to the appropriate center predicated on the PMOA of the combined product, in order to ensure clear regulatory responsibilities and coordinated evaluation processes [292,294,295].

Ultrasound, to deliver genes non-invasively, offers safer and more targeted delivery than alternative methods involving magnetic, light, or electric fields. By using ultrasound contrast agents (UCAs), such as microbubbles (MBs), enables both imaging and ultrasound-mediated disease treatment. For example, drug-carrying MBs circulate in the bloodstream, specifically targeting certain regional sites and releasing drugs at those sites. This approach enhances our comprehension of elucidating the mechanisms of disease and unlocks new avenues for diagnosis and treatment [296]. UCAs can be developed into various forms, including nanobubbles, echo liposomes, and nanodroplets, which have biocompatibility and stability, capable of targeting blood vessels and tissues [[297], [298], [299]]. Moreover, therapeutic gases like nitric oxide (NO), carbon monoxide (CO), and hydrogen sulfide (H_2_S) are being investigated. These therapeutic gases not only function as UCAs but also enhance the effects of therapeutic, representing an exciting direction for future contrast agents [300]. Recent advances in materials chemistry have made it possible to deliver drugs or genes in a highly controlled manner—spatially, temporally, and in specific doses. Novel multifunctional nanotherapeutic agents, combined with responsive ultrasound imaging, can target tumor accumulation and guide ablation functions [301]. By conjugating antibodies or ligands, MBs can target disease-related markers present on endothelial cells or other target cells, such as the vascular endothelial growth factor receptor 2 (VEGFR2), for improved targeting efficiency [302,303]. Additionally, injectable MBs loaded with enzymes and nanoparticles can regulate cell homeostasis through enzymatic reactions, controlling the targeted release of substances in both space and time to achieve therapeutic effects [304]. Ultrasound- and MB-mediated drug delivery has demonstrated its ability to facilitate efficient delivery of drugs and genes delivery to specific tissues while limiting systemic doses and adverse effect [305]. The safety and efficacy of focused ultrasound (FUS)-mediated drug and gene delivery are strongly affected by acoustic parameters, including ultrasound treatment duration, pulse repetition rate, pulse length, and MB concentration. Among these, acoustic pressure and MB concentration are critical determinants in blood–brain barrier (BBB) opening [306]. High-intensity FUS pressure has the potential to induce damage to the vascular wall, hemorrhaging, ischemic events, apoptosis, and inflammatory reactions, overly high MB concentrations may result in excessive BBB opening and associated damage [307,308].

Despite the promising potential, ultrasound-mediated gene therapy still faces significant challenges, including gene transfer efficiency, specificity, safety, and duration of gene expression. Possible immune reactions induced by nanoparticles must also be carefully considered [309]. The clinical trial design of ultrasound-mediated gene therapy has numerous limitations: Firstly, the definition of “dose” is complex, as it needs to take into account the quantity of the gene vector, the physical parameters of ultrasound (sound pressure, frequency, etc.) and the characteristics of microbubbles, and the optimal combination window is difficult to determine; Secondly, individual differences in patients (such as skull thickness, pathological state of the target tissue) will significantly influence the distribution of ultrasound energy and the efficiency of gene delivery, which poses high requirements for the inclusion criteria of the subjects and the consistency of the results; Furthermore, setting up an appropriate control group (such as sham ultrasound irradiation) has ethical and practical difficulties, and long-term safety and the persistence of gene expression follow-up are of crucial importance [[310], [311], [312], [313]]. Meanwhile, this technology, as a Combination Product, faces severe regulatory hurdles. It integrates biological products (genetic vectors), devices (ultrasound equipment), and drugs (microbubbles) all in one, and needs to meet the strict regulations of all three at the same time. This makes the chemical, production and control (CMC) process extremely complex. Regulatory agencies are concerned about its unique risks, such as mechanical damage to the target tissue caused by ultrasound, off-target gene expression, and immune activation effects. Therefore, they usually require detailed non-clinical safety data based on large animal models [[314], [315], [316], [317], [318]]. Currently, there are no specific review guidelines for this technology, and sponsors need to have extensive preliminary communication with regulatory agencies to jointly explore feasible development and approval paths. Overcoming these challenges urgently requires close interdisciplinary cooperation and innovation. However, with increasing clinical successes and regulatory approvals, ultrasound-mediated gene therapy is gaining momentum. Researchers remain cautiously optimistic that effective, long-lasting, and safe therapies will bring significant benefits to patients.

## CRediT authorship contribution statement

**Jie Wu:** Writing – review & editing, Writing – original draft. **Yang Gao:** Writing – review & editing. **Hongju Zhou:** Writing – review & editing. **Zhengbao Yang:** Writing – review & editing. **Raul D. Rodriguez:** Writing – review & editing. **Xiaohui Xu:** Writing – review & editing, Supervision, Investigation, Conceptualization. **Lang Ma:** Writing – review & editing, Supervision, Methodology, Conceptualization. **Li Qiu:** Writing – review & editing, Supervision, Methodology, Conceptualization.

## Declaration of competing interest

The authors declare that they have no known competing financial interests or personal relationships that could have appeared to influence the work reported in this paper.

## References

[1] Feng Z., Xiang X., Huang J., Wang L., Zhu B., Zhou H., Pang H., Cheng C., Ma L., Qiu L. (2023). Intelligent sonocatalytic nanoagents for energy conversion-based therapies. Adv. Func. Mater..

[2] Zuo M., Xiao R., Du F., Cheng C., Rodriguez R.D., Ma L., Zhu B., Qiu L. (2024). Ultrasound-activated mechanochemical reactions for controllable biomedical applications. Smart Mater. Med..

[3] Sigrist R.M.S., Liau J., Kaffas A.E., Chammas M.C., Willmann J.K. (2017). Ultrasound elastography: review of techniques and clinical applications. Theranostics.

[4] Huang D., Wang J., Song C., Zhao Y. (2023). Ultrasound-responsive matters for biomedical applications. Innovation (Camb)..

[5] Moyano D.B., Paraiso D.A., González-Lezcano R.A. (2022). Possible effects on health of ultrasound exposure, risk factors in the work environment and occupational safety review. Healthcare (Basel).

[6] Rong X., Tang Y., Cao S., Xiao S., Wang H., Zhu B., Huang S., Adeli M., Rodriguez R.D., Cheng C., Ma L., Qiu L. (2023). An extracellular vesicle-cloaked multifaceted biocatalyst for ultrasound-augmented tendon matrix reconstruction and immune microenvironment regulation. ACS Nano.

[7] Liao M., Du J., Chen L., Huang J., Yang R., Bao W., Zeng K., Wang W., Aphan B.C., Wu Z., Ma L., Lu Q. (2024). Sono-activated materials for enhancing focused ultrasound ablation: Design and application in biomedicine. Acta Biomater..

[8] Wang L., Zhang L., Chen F., Li Q., Zhu B., Tang Y., Yang Z., Cheng C., Qiu L., Ma L. (2024). Polymerized network-based artificial peroxisome reprogramming macrophages for photoacoustic imaging-guided treatment of rheumatoid arthritis. ACS Appl. Mater. Interfaces.

[9] Huang X., Zhou H., Lv N., Zhao Z., Tian T., Huang J., Rodriguez R.D., Luo H., Cheng C., Ma L. (2024). Immunomodulating sonocatalytic nanoagents with dual-functional Ir-N centers and narrow bandgap for reversing immunosuppression and potentiating ovarian cancer immunotherapy. Adv. Func. Mater..

[10] Liao M., Zhang Q., Huang J., Huang X., Cheng C., Tu J., Zhang D., Lu Q., Ma L. (2024). Near-infrared and ultrasound triggered Pt/Pd-engineered cluster bombs for the treatment of solid tumors. J. Control. Release.

[11] Wirth T., Parker N., Ylä-Herttuala S. (2013). History of gene therapy. Gene.

[12] Li H., Yang Y., Hong W., Huang M., Wu M., Zhao X. (2020). Applications of genome editing technology in the targeted therapy of human diseases: mechanisms, advances and prospects. Signal Transduct. Target. Ther..

[13] Bulcha J.T., Wang Y., Ma H., Tai P.W.L., Gao G. (2021). Viral vector platforms within the gene therapy landscape. Signal Transduct. Target. Ther..

[14] Cring M.R., Sheffield V.C. (2022). Gene therapy and gene correction: targets, progress, and challenges for treating human diseases. Gene Ther..

[15] Sayed N., Allawadhi P., Khurana A., Singh V., Navik U., Pasumarthi S.K., Khurana I., Banothu A.K., Weiskirchen R., Bharani K.K. (2022). Gene therapy: comprehensive overview and therapeutic applications. Life Sci..

[16] He Y., Li D., Wu L., Yin X., Zhang X., Patterson L.H., Zhang J. (2023). Metal-organic frameworks for gene therapy and detection. Adv. Func. Mater..

[17] Zhang Q., Kuang G., Li W., Wang J., Ren H., Zhao Y. (2023). Stimuli-responsive gene delivery nanocarriers for cancer therapy. Nano Micro Lett..

[18] Li X., Le Y., Zhang Z., Nian X., Liu B., Yang X. (2023). Viral vector-based gene therapy. Int. J. Mol. Sci..

[19] Wang C., Pan C., Yong H., Wang F., Bo T., Zhao Y., Ma B., He W., Li M. (2023). Emerging non-viral vectors for gene delivery. J. Nanobiotechnol..

[20] Perry C., Rayat A. (2021). Lentiviral vector bioprocessing. Viruses.

[21] Kanerva A., Hemminki A. (2004). Modified adenoviruses for cancer gene therapy. Int. J. Cancer.

[22] Ertl H.C.J. (2022). Immunogenicity and toxicity of AAV gene therapy. Front. Immunol..

[23] Campelo S.N., Huang P.H., Buie C.R., Davalos R.V. (2023). Recent Advancements in Electroporation Technologies: from Bench to Clinic. Annu. Rev. Biomed. Eng..

[24] Zhang R., Zhao J., Li L. (2022). Growth inhibition of Trichophyton rubrum by laser irradiation: exploring further experimental aspects in an in vitro evaluation study. BMC Microbiol..

[25] Zhang Y., Yu L.C. (2008). Microinjection as a tool of mechanical delivery. Curr. Opin. Biotechnol..

[26] Xie A., Belcik T., Qi Y., Morgan T.K., Champaneri S.A., Taylor S., Davidson B.P., Zhao Y., Klibanov A.L., Kuliszewski M.A., Leong-Poi H., Ammi A., Lindner J.R. (2012). Ultrasound-mediated vascular gene transfection by cavitation of endothelial-targeted cationic microbubbles. JACC: Cardiovasc Imaging.

[27] Wu Y., Wang L., Xiong Y., Zhou Q., Li L., Chen G., Ping Y., Davidson G., Levkin P.A., Gao L., Deng W. (2020). Cell-based high-throughput screening of cationic polymers for efficient DNA and siRNA delivery. Acta Biomater..

[28] Semple S.C., Akinc A., Chen J., Sandhu A.P., Mui B.L., Cho C.K., Sah D.W., Stebbing D., Crosley E.J., Yaworski E., Hafez I.M., Dorkin J.R., Qin J., Lam K., Rajeev K.G., Wong K.F., Jeffs L.B., Nechev L., Eisenhardt M.L., Jayaraman M., Kazem M., Maier M.A., Srinivasulu M., Weinstein M.J., Chen Q., Alvarez R., Barros S.A., De S., Klimuk S.K., Borland T., Kosovrasti V., Cantley W.L., Tam Y.K., Manoharan M., Ciufolini M.A., Tracy M.A., de Fougerolles A., MacLachlan I., Cullis P.R., Madden T.D., Hope M.J. (2010). Rational design of cationic lipids for siRNA delivery. Nat. Biotechnol..

[29] Zohra F.T., Chowdhury E.H., Akaike T. (2009). High performance mRNA transfection through carbonate apatite-cationic liposome conjugates. Biomaterials.

[30] Turali-Emre E.S., Emre A.E., Vecchio D.A., Kadiyala U., VanEpps J.S., Kotov N.A. (2023). Self-Organization of Iron sulfide nanoparticles into complex multicompartment supraparticles. Adv. Mater..

[31] Zu H., Gao D. (2021). Non-viral vectors in gene therapy: recent development, challenges, and prospects. AAPS J..

[32] Shokouhi A.R., Chen Y., Yoh H.Z., Brenker J., Alan T., Murayama T., Suu K., Morikawa Y., Voelcker N.H., Elnathan R. (2023). Engineering efficient CAR-T cells via electroactive nanoinjection. Adv. Mater..

[33] Campillo-Davo D., De Laere M., Roex G., Versteven M., Flumens D., Berneman Z.N., Van Tendeloo V.F.I., Anguille S., Lion E. (2021). The ins and outs of messenger RNA electroporation for physical gene delivery in immune cell-based therapy. Pharmaceutics.

[34] Liu X., Rong N., Tian Z., Rich J., Niu L., Li P., Huang L., Dong Y., Zhou W., Zhang P., Chen Y., Wang C., Meng L., Huang T.J., Zheng H. (2024). Acoustothermal transfection for cell therapy. Sci. Adv..

[35] Davis A.A., Farrar M.J., Nishimura N., Jin M.M., Schaffer C.B. (2013). Optoporation and genetic manipulation of cells using femtosecond laser pulses. Biophys. J ..

[36] Chow Y.T., Chen S., Wang R., Liu C., Kong C.W., Li R.A., Cheng S.H., Sun D. (2016). Single cell transfection through precise microinjection with quantitatively controlled injection volumes. Sci. Rep..

[37] Wang T., Zhao H., Jing S., Fan Y., Sheng G., Ding Q., Liu C., Wu H., Liu Y. (2023). Magnetofection of miR-21 promoted by electromagnetic field and iron oxide nanoparticles via the p38 MAPK pathway contributes to osteogenesis and angiogenesis for intervertebral fusion. J. Nanobiotechnol..

[38] Duan L., Yang L., Jin J., Yang F., Liu D., Hu K., Wang Q., Yue Y., Gu N. (2020). Micro/nano-bubble-assisted ultrasound to enhance the EPR effect and potential theranostic applications. Theranostics.

[39] Zhao C., Pan Y., Yu G., Zhao X.-Z., Chen X., Rao L. (2023). Vesicular antibodies: shedding light on antibody therapeutics with cell membrane nanotechnology. Adv. Mater..

[40] Shokouhi A.-R., Chen Y., Yoh H.Z., Brenker J., Alan T., Murayama T., Suu K., Morikawa Y., Voelcker N.H., Elnathan R. (2023). Engineering efficient CAR-T cells via electroactive nanoinjection. Adv. Mater..

[41] Pan J., Chiang C.-L., Wang X., Bertani P., Ma Y., Cheng J., Talesara V., Lee L.J., Lu W. (2023). Cell membrane damage and cargo delivery in nano-electroporation. Nanoscale.

[42] Ivanovski F., Meško M., Lebar T., Rupnik M., Lainšček D., Gradišek M., Jerala R., Benčina M. (2024). Ultrasound-mediated spatial and temporal control of engineered cells in vivo. Nat. Commun..

[43] Liu P., Foiret J., Situ Y., Zhang N., Kare A.J., Wu B., Raie M.N., Ferrara K.W., Qi L.S. (2023). Sonogenetic control of multiplexed genome regulation and base editing. Nat. Commun..

[44] Shi Y., Weng W., Chen M., Huang H., Chen X., Peng Y., Hu Y. (2023). Improving DNA vaccination performance through a new microbubble design and an optimized sonoporation protocol. Ultrason. Sonochem..

[45] Chen J., Wang B., Wang Y., Radermacher H., Qi J., Momoh J., Lammers T., Shi Y., Rix A., Kiessling F. (2024). mRNA sonotransfection of tumors with polymeric microbubbles: co-formulation versus co-administration. Adv. Sci..

[46] Li Y., Wu P., Zhu M., Liang M., Zhang L., Zong Y., Wan M. (2023). High-performance delivery of a CRISPR Interference system via lipid-polymer hybrid nanoparticles combined with ultrasound-mediated microbubble destruction for tumor-specific gene repression. Adv. Healthc. Mater..

[47] Son S., Kim J.H., Wang X., Zhang C., Yoon S.A., Shin J., Sharma A., Lee M.H., Cheng L., Wu J., Kim J.S. (2020). Multifunctional sonosensitizers in sonodynamic cancer therapy. Chem. Soc. Rev..

[48] Wu T., Liu Y., Cao Y., Liu Z. (2022). Engineering macrophage exosome disguised biodegradable nanoplatform for enhanced sonodynamic therapy of glioblastoma. Adv. Mater..

[49] Xu Q., Zhan G., Zhang Z., Yong T., Yang X., Gan L. (2021). Manganese porphyrin-based metal-organic framework for synergistic sonodynamic therapy and ferroptosis in hypoxic tumors. Theranostics.

[50] Xu P.Y., Kumar Kankala R., Wang S.B., Chen A.Z. (2023). Sonodynamic therapy-based nanoplatforms for combating bacterial infections. Ultrason. Sonochem..

[51] Liu P., Wang Z., Ou X., Wu P., Zhang Y., Wu S., Xiao X., Li Y., Ye F., Tang H. (2022). The FUS/circEZH2/KLF5/ feedback loop contributes to CXCR4-induced liver metastasis of breast cancer by enhancing epithelial-mesenchymal transition. Mol. Cancer.

[52] Wang J., Li Z., Pan M., Fiaz M., Hao Y., Yan Y., Sun L., Yan F. (2022). Ultrasound-mediated blood-brain barrier opening: an effective drug delivery system for theranostics of brain diseases. Adv. Drug Deliv. Rev..

[53] Lu D., Wang L., Wang L., An L., Huo M., Xu H., Shi J. (2022). Probiotic engineering and targeted sonoimmuno-therapy augmented by STING agonist. Adv. Sci. (Weinh).

[54] El Kadi S., Porter T.R., Verouden N.J.W., van Rossum A.C., Kamp O. (2022). Contrast ultrasound, sonothrombolysis and sonoperfusion in cardiovascular disease: shifting to theragnostic clinical trials. JACC: Cardiovasc. Imaging.

[55] Cai K., Jiao Y., Quan Q., Hao Y., Liu J., Wu L. (2021). Improved activity of MC3T3-E1 cells by the exciting piezoelectric BaTiO(3)/TC4 using low-intensity pulsed ultrasound. Bioact. Mater..

[56] Zheng Y., Li C., Zhang C., He J., Jiang X., Ta D. (2023). Distinct thermal effect on biological tissues using subwavelength ultrasound metalens at megahertz. iScience.

[57] Liu Y., Yu W., Liu Y. (2019). Effect of ultrasound on dissolution of Al in Sn. Ultrason. Sonochem..

[58] Deckers R., Moonen C.T.W. (2010). Ultrasound triggered, image guided, local drug delivery. J. Control. Release.

[59] Vujaskovic Z., Poulson J.M., Gaskin A.A., Thrall D.E., Page R.L., Charles H.C., MacFall J.R., Brizel D.M., Meyer R.E., Prescott D.M., Samulski T.V., Dewhirst M.W. (2000). Temperature-dependent changes in physiologic parameters of spontaneous canine soft tissue sarcomas after combined radiotherapy and hyperthermia treatment. Int. J. Radiat. Oncol. Biol. Phys..

[60] Karino T., Koga S., Maeta M. (1988). Experimental studies of the effects of local hyperthermia on blood flow, oxygen pressure and pH in tumors. Jpn. J. Surg..

[61] Gaber M.H., Wu N.Z., Hong K., Huang S.K., Dewhirst M.W., Papahadjopoulos D. (1996). Thermosensitive liposomes: extravasation and release of contents in tumor microvascular networks. Int. J. Radiat. Oncol. Biol. Phys..

[62] Foley J.L., Eames M., Snell J., Hananel A., Kassell N., Aubry J.F. (2013). Image-guided focused ultrasound: state of the technology and the challenges that lie ahead. Imaging Med..

[63] Wu Y., Liu Y., Huang Z., Wang X., Jin Z., Li J., Limsakul P., Zhu L., Allen M., Pan Y., Bussell R., Jacobson A., Liu T., Chien S., Wang Y. (2021). Control of the activity of CAR-T cells within tumours via focused ultrasound. Nat. Biomed. Eng..

[64] Zhang N., Wang J., Foiret J., Dai Z., Ferrara K.W. (2021). Synergies between therapeutic ultrasound, gene therapy and immunotherapy in cancer treatment. Adv. Drug Deliv. Rev..

[65] Walsh A.P.G., Gordon H.N., Peter K., Wang X. (2021). Ultrasonic particles: an approach for targeted gene delivery. Adv. Drug Deliv. Rev..

[66] Prentice P., Cuschieri A., Dholakia K., Prausnitz M., Campbell P. (2005). Membrane disruption by optically controlled microbubble cavitation. Nat. Phys..

[67] Humphrey V.F. (2007). Ultrasound and matter–physical interactions. Prog. Biophys. Mol. Biol..

[68] Fant C., Lafond M., Rogez B., Castellanos I.S., Ngo J., Mestas J.L., Padilla F., Lafon C. (2019). In vitro potentiation of doxorubicin by unseeded controlled non-inertial ultrasound cavitation. Sci. Rep..

[69] Yudina A., Lepetit-Coiffé M., Moonen C.T. (2011). Evaluation of the temporal window for drug delivery following ultrasound-mediated membrane permeability enhancement. Mol. Imaging Biol..

[70] Ilovitsh T., Feng Y., Foiret J., Kheirolomoom A., Zhang H., Ingham E.S., Ilovitsh A., Tumbale S.K., Fite B.Z., Wu B., Raie M.N., Zhang N., Kare A.J., Chavez M., Qi L.S., Pelled G., Gazit D., Vermesh O., Steinberg I., Gambhir S.S., Ferrara K.W. (2020). Low-frequency ultrasound-mediated cytokine transfection enhances T cell recruitment at local and distant tumor sites. PNAS.

[71] Zhao X., Wright A., Goertz D.E. (2023). An optical and acoustic investigation of microbubble cavitation in small channels under therapeutic ultrasound conditions. Ultrason. Sonochem..

[72] Apfel R.E. (1982). Acoustic cavitation: a possible consequence of biomedical uses of ultrasound. Br. J. Cancer Suppl..

[73] Sboros V. (2008). Response of contrast agents to ultrasound. Adv. Drug Deliv. Rev..

[74] Deckers R., Rome C., Moonen C.T. (2008). The role of ultrasound and magnetic resonance in local drug delivery. J. Magn. Reson..

[75] Kooiman K., Roovers S., Langeveld S.A.G., Kleven R.T., Dewitte H., O'Reilly M.A., Escoffre J.M., Bouakaz A., Verweij M.D., Hynynen K., Lentacker I., Stride E., Holland C.K. (2020). Ultrasound-responsive cavitation nuclei for therapy and drug delivery. Ultrasound Med. Biol..

[76] Bader K.B., Vlaisavljevich E., Maxwell A.D. (2019). For whom the bubble grows: physical principles of bubble nucleation and dynamics in histotripsy ultrasound therapy. Ultrasound Med. Biol..

[77] Vlaisavljevich E., Lin K.W., Maxwell A., Warnez M.T., Mancia L., Singh R., Putnam A.J., Fowlkes B., Johnsen E., Cain C., Xu Z. (2015). Effects of ultrasound frequency and tissue stiffness on the histotripsy intrinsic threshold for cavitation. Ultrasound Med. Biol..

[78] Padilla F., Brenner J., Prada F., Klibanov A.L. (2023). Theranostics in the vasculature: bioeffects of ultrasound and microbubbles to induce vascular shutdown. Theranostics.

[79] Hu Z., Zhang H., Mordovanakis A., Paulus Y.M., Liu Q., Wang X., Yang X. (2017). High-precision, non-invasive anti-microvascular approach via concurrent ultrasound and laser irradiation. Sci. Rep..

[80] Kramer J.F. (1984). Ultrasound: evaluation of its mechanical and thermal effects. Arch. Phys. Med. Rehabil..

[81] Xiang H., Chen Y. (2019). Energy-converting nanomedicine. Small.

[82] Huang H., Zheng Y., Chang M., Song J., Xia L., Wu C., Jia W., Ren H., Feng W., Chen Y. (2024). Ultrasound-based micro-/nanosystems for biomedical applications. Chem. Rev..

[83] Anguela X.M., High K.A. (2019). Entering the modern era of gene therapy. Annu. Rev. Med..

[84] Jeon Y.H., Jung Y.T. (2022). Production of a replicating retroviral vector expressing Reovirus fast protein for cancer gene therapy. J. Virol. Methods.

[85] Fus-Kujawa A., Prus P., Bajdak-Rusinek K., Teper P., Gawron K., Kowalczuk A., Sieron A.L. (2021). An overview of methods and tools for transfection of eukaryotic cells in vitro. Front. Bioeng. Biotechnol..

[86] Ricobaraza A., Gonzalez-Aparicio M., Mora-Jimenez L., Lumbreras S., Hernandez-Alcoceba R. (2020). High-capacity adenoviral vectors: expanding the scope of gene therapy. Int. J. Mol. Sci..

[87] Yari A., Bamdad T., Hosseini S.Y. (2023). Comparison of three different methods of transfection for the production of recombinant adenovirus expressing human carcinoembryonic antigen gene. Arch. Razi Inst..

[88] Mével M., Bouzelha M., Leray A., Pacouret S., Guilbaud M., Penaud-Budloo M., Alvarez-Dorta D., Dubreil L., Gouin S.G., Combal J.P., Hommel M., Gonzalez-Aseguinolaza G., Blouin V., Moullier P., Adjali O., Deniaud D., Ayuso E. (2019). Chemical modification of the adeno-associated virus capsid to improve gene delivery. Chem. Sci..

[89] Moço P.D., Dash S., Kamen A.A. (2024). Enhancement of adeno-associated virus serotype 6 transduction into T cells with cell-penetrating peptides. J. Gene Med..

[90] Page A., Fusil F., Cosset F.L. (2020). Toward Tightly Tuned Gene Expression following Lentiviral Vector Transduction. Viruses.

[91] Douka S., Brandenburg L.E., Casadidio C., Walther J., Garcia B.B.M., Spanholtz J., Raimo M., Hennink W.E., Mastrobattista E., Caiazzo M. (2023). Lipid nanoparticle-mediated messenger RNA delivery for ex vivo engineering of natural killer cells. J. Control. Release.

[92] Billingsley M.M., Gong N., Mukalel A.J., Thatte A.S., El-Mayta R., Patel S.K., Metzloff A.E., Swingle K.L., Han X., Xue L., Hamilton A.G., Safford H.C., Alameh M.G., Papp T.E., Parhiz H., Weissman D., Mitchell M.J. (2024). In vivo mRNA CAR T cell engineering via targeted ionizable lipid nanoparticles with extrahepatic tropism. Small.

[93] Olden B.R., Cheng Y., Yu J.L., Pun S.H. (2018). Cationic polymers for non-viral gene delivery to human T cells. J. Control. Release.

[94] Suberi A., Grun M.K., Mao T., Israelow B., Reschke M., Grundler J., Akhtar L., Lee T., Shin K., Piotrowski-Daspit A.S., Homer R.J., Iwasaki A., Suh H.W., Saltzman W.M. (2023). Polymer nanoparticles deliver mRNA to the lung for mucosal vaccination. Sci. Transl. Med..

[95] Wang C., Du L., Zhou J., Meng L., Cheng Q., Wang C., Wang X., Zhao D., Huang Y., Zheng S., Cao H., Zhang J., Deng L., Liang Z., Dong A. (2017). Elaboration on the distribution of hydrophobic segments in the chains of amphiphilic cationic polymers for small interfering RNA delivery. ACS Appl. Mater. Interfaces.

[96] Liu Y., Wang T., He F., Liu Q., Zhang D., Xiang S., Su S., Zhang J. (2011). An efficient calcium phosphate nanoparticle-based nonviral vector for gene delivery. Int. J. Nanomed..

[97] Qiu C., Wei W., Sun J., Zhang H.T., Ding J.S., Wang J.C., Zhang Q. (2016). Systemic delivery of siRNA by hyaluronan-functionalized calcium phosphate nanoparticles for tumor-targeted therapy. Nanoscale.

[98] Zhou Q., Wang Y., Xiang J., Piao Y., Zhou Z., Tang J., Liu X., Shen Y. (2018). Stabilized calcium phosphate hybrid nanocomposite using a benzoxaborole-containing polymer for pH-responsive siRNA delivery. Biomater. Sci..

[99] Yu J., Chen Z., Yan F. (2019). Advances in mechanism studies on ultrasonic gene delivery at cellular level. Prog. Biophys. Mol. Biol..

[100] Yoshida M., Kawakami S., Kono Y., Un K., Higuchi Y., Maruyama K., Yamashita F., Hashida M. (2014). Enhancement of the anti-tumor effect of DNA vaccination using an ultrasound-responsive mannose-modified gene carrier in combination with doxorubicin-encapsulated PEGylated liposomes. Int. J. Pharm..

[101] Seyed Jafari S.M., Blank F., Ramser H.E., Woessner A.E., Shafighi M., Geiser T., Quinn K.P., Hunger R.E., Gazdhar A. (2021). Efficacy of combined in-vivo electroporation-mediated gene transfer of VEGF, HGF, and IL-10 on skin flap survival, monitored by label-free optical imaging: a feasibility study. Front. Surg..

[102] VanderBurgh J.A., Corso G.T., Levy S.L., Craighead H.G. (2024). A multiplexed microfluidic continuous-flow electroporation system for efficient cell transfection. Biomed. Microdevices.

[103] Kumar A.R.K., Shou Y., Chan B., K.L, Tay A. (2021). Materials for improving immune cell transfection. Adv. Mater..

[104] Jo D.H., Kaczmarek S., Shin O., Wang L., Cowan J., McComb S., Lee S.H. (2023). Simultaneous engineering of natural killer cells for CAR transgenesis and CRISPR-Cas9 knockout using retroviral particles. Mol. Ther. Methods Clin. Dev..

[105] Khalifehzadeh R., Arami H. (2020). Biodegradable calcium phosphate nanoparticles for cancer therapy. Adv. Colloid Interface Sci..

[106] Rajabzadeh A., Hamidieh A.A., Rahbarizadeh F. (2021). Spinoculation and retronectin highly enhance the gene transduction efficiency of Mucin-1-specific chimeric antigen receptor (CAR) in human primary T cells. BMC Mol. Cell Biol..

[107] Kantor A., McClements M.E., MacLaren R.E. (2020). CRISPR-Cas9 DNA base-editing and prime-editing. Int. J. Mol. Sci..

[108] Srivastava A., Mallela K.M.G., Deorkar N., Brophy G. (2021). Manufacturing challenges and rational formulation development for AAV viral vectors. J. Pharm. Sci..

[109] Guimaraes T.A.C., Georgiou M., Bainbridge J.W.B., Michaelides M. (2021). Gene therapy for neovascular age-related macular degeneration: rationale, clinical trials and future directions. Br. J. Ophthalmol..

[110] Lundstrom K. (2020). Viral vectors applied for RNAi-based antiviral therapy. Viruses.

[111] Rauch-Wirth L., Renner A., Kaygisiz K., Weil T., Zimmermann L., Rodriguez-Alfonso A.A., Schütz D., Wiese S., Ständker L., Weil T., Schmiedel D., Münch J. (2023). Optimized peptide nanofibrils as efficient transduction enhancers for in vitro and ex vivo gene transfer. Front. Immunol..

[112] Gautam M., Jozic A., Su G.L., Herrera-Barrera M., Curtis A., Arrizabalaga S., Tschetter W., Ryals R.C., Sahay G. (2023). Lipid nanoparticles with PEG-variant surface modifications mediate genome editing in the mouse retina. Nat. Commun..

[113] Boloix A., Feiner-Gracia N., Köber M., Repetto J., Pascarella R., Soriano A., Masanas M., Segovia N., Vargas-Nadal G., Merlo-Mas J., Danino D., Abutbul-Ionita I., Foradada L., Roma J., Córdoba A., Sala S., de Toledo J.S., Gallego S., Veciana J., Albertazzi L., Segura M.F., Ventosa N. (2022). Engineering pH-sensitive stable nanovesicles for delivery of MicroRNA therapeutics. Small.

[114] Li Z., Amaya L., Pi R., Wang S.K., Ranjan A., Waymouth R.M., Blish C.A., Chang H.Y., Wender P.A. (2023). Charge-altering releasable transporters enhance mRNA delivery in vitro and exhibit in vivo tropism. Nat. Commun..

[115] Parayath N.N., Stephan S.B., Koehne A.L., Nelson P.S., Stephan M.T. (2020). In vitro-transcribed antigen receptor mRNA nanocarriers for transient expression in circulating T cells in vivo. Nat. Commun..

[116] Olton D.Y., Close J.M., Sfeir C.S., Kumta P.N. (2011). Intracellular trafficking pathways involved in the gene transfer of nano-structured calcium phosphate-DNA particles. Biomaterials.

[117] Klesing J., Wiehe A., Gitter B., Gräfe S., Epple M. (2010). Positively charged calcium phosphate/polymer nanoparticles for photodynamic therapy. J. Mater. Sci. - Mater. Med..

[118] Choi K.Y., Silvestre O.F., Huang X., Min K.H., Howard G.P., Hida N., Jin A.J., Carvajal N., Lee S.W., Hong J.I., Chen X. (2014). Versatile RNA interference nanoplatform for systemic delivery of RNAs. ACS Nano.

[119] Bellary A., Nowak C., Iwanicki I., Flores-Guzman F., Wu L., Kandel J.J., Laetsch T.W., Bleris L., Hernandez S.L., Sirsi S.R. (2023). Non-viral nitric oxide-based gene therapy improves perfusion and liposomal doxorubicin sonopermeation in neuroblastoma models. Theranostics.

[120] Mishel S., Shneyer B., Korsensky L., Goldshmidt-Tran O., Haber T., Machluf M., Ron D. (2017). Delivery of the gene encoding the tumor suppressor Sef into prostate tumors by therapeutic-ultrasound inhibits both tumor angiogenesis and growth. Sci. Rep..

[121] Bouakaz A., Zeghimi A., Doinikov A.A. (2016). Sonoporation: concept and mechanisms. Adv. Exp. Med. Biol..

[122] Kooiman K., Vos H.J., Versluis M., de Jong N. (2014). Acoustic behavior of microbubbles and implications for drug delivery. Adv. Drug Deliv. Rev..

[123] Tachibana K., Uchida T., Ogawa K., Yamashita N., Tamura K. (1999). Induction of cell-membrane porosity by ultrasound. Lancet.

[124] Mehier-Humbert S., Bettinger T., Yan F., Guy R.H. (2005). Plasma membrane poration induced by ultrasound exposure: implication for drug delivery. J. Control. Release.

[125] León G., Martínez G., Guzmán M.A., Moreno J.I., Miguel B., Fernández-López J.A. (2013). Increasing stability and transport efficiency of supported liquid membranes through a novel ultrasound-assisted preparation method. Its application to cobalt(II) removal. Ultrason. Sonochem..

[126] Nishimura K., Yonezawa K., Fumoto S., Miura Y., Hagimori M., Nishida K., Kawakami S. (2019). Application of direct sonoporation from a defined surface area of the peritoneum: evaluation of transfection characteristics in mice. Pharmaceutics.

[127] Newman C.M., Bettinger T. (2007). Gene therapy progress and prospects: ultrasound for gene transfer. Gene Ther..

[128] Zolghadrnasab M., Mousavi A., Farmany A., Arpanaei A. (2021). Ultrasound-mediated gene delivery into suspended plant cells using polyethyleneimine-coated mesoporous silica nanoparticles. Ultrason. Sonochem..

[129] Király A., Farkas D., Dobránszki J. (2025). Ultrasound in plant life and its application perspectives in horticulture and agriculture. Horticulturae.

[130] Fan Z., Kumon R.E., Deng C.X. (2014). Mechanisms of microbubble-facilitated sonoporation for drug and gene delivery. Ther. Deliv..

[131] Beekers I., Vegter M., Lattwein K.R., Mastik F., Beurskens R., van der Steen A.F.W., de Jong N., Verweij M.D., Kooiman K. (2020). Opening of endothelial cell-cell contacts due to sonoporation. J. Control. Release.

[132] Izadifar Z., Babyn P., Chapman D. (2017). Mechanical and biological effects of ultrasound: a review of present knowledge. Ultrasound Med. Biol..

[133] Arvanitis C.D., Askoxylakis V., Guo Y., Datta M., Kloepper J., Ferraro G.B., Bernabeu M.O., Fukumura D., McDannold N., Jain R.K. (2018). Mechanisms of enhanced drug delivery in brain metastases with focused ultrasound-induced blood-tumor barrier disruption. PNAS.

[134] Sheikov N., McDannold N., Sharma S., Hynynen K. (2008). Effect of focused ultrasound applied with an ultrasound contrast agent on the tight junctional integrity of the brain microvascular endothelium. Ultrasound Med. Biol..

[135] Helfield B., Chen X., Watkins S.C., Villanueva F.S. (2016). Biophysical insight into mechanisms of sonoporation. PNAS.

[136] Liu W.W., Huang S.H., Li P.C. (2019). Synchronized optical and acoustic droplet vaporization for effective sonoporation. Pharmaceutics.

[137] Hauff P., Seemann S., Reszka R., Schultze-Mosgau M., Reinhardt M., Buzasi T., Plath T., Rosewicz S., Schirner M. (2005). Evaluation of gas-filled microparticles and sonoporation as gene delivery system: feasibility study in rodent tumor models. Radiology.

[138] Escoffre J.M., Zeghimi A., Novell A., Bouakaz A. (2013). In-vivo gene delivery by sonoporation: recent progress and prospects. Curr. Gene Ther..

[139] Fan Z., Liu H., Mayer M., Deng C.X. (2012). Spatiotemporally controlled single cell sonoporation. PNAS.

[140] Fan Z., Xue X., Fu J., Deng C.X. (2020). Visualization and quantification of dynamic intercellular coupling in human embryonic stem cells using single cell sonoporation. Sci. Rep..

[141] Marmottant P., Hilgenfeldt S. (2003). Controlled vesicle deformation and lysis by single oscillating bubbles. Nature.

[142] Zhang N., Foiret J., Kheirolomoom A., Liu P., Feng Y., Tumbale S., Raie M., Wu B., Wang J., Fite B.Z., Dai Z., Ferrara K.W. (2021). Optimization of microbubble-based DNA vaccination with low-frequency ultrasound for enhanced cancer immunotherapy. Adv Ther (weinh)..

[143] Ikeda-Dantsuji F.Y. (2008). Sonodynamic therapy. Ultrasonics.

[144] Hiraoka W., Honda H., Feril L.B., Kudo N., Kondo T. (2006). Comparison between sonodynamic effect and photodynamic effect with photosensitizers on free radical formation and cell killing. Ultrason. Sonochem..

[145] Mason T.J., Cobley A.J., Graves J.E., Morgan D. (2011). New evidence for the inverse dependence of mechanical and chemical effects on the frequency of ultrasound. Ultrason. Sonochem..

[146] Dehghani M.H., Karri R.R., Koduru J.R., Manickam S., Tyagi I., Mubarak N.M. (2023). Suhas, recent trends in the applications of sonochemical reactors as an advanced oxidation process for the remediation of microbial hazards associated with water and wastewater: a critical review. Ultrason. Sonochem..

[147] Zhang Q., Bao C., Cai X., Jin L., Sun L., Lang Y., Li L. (2018). Sonodynamic therapy-assisted immunotherapy: a novel modality for cancer treatment. Cancer Sci..

[148] Feng Z., Yao Y., Wang Z., Xiang X., Wang L., Xiao X., Tang Y., Hu W., Qiu L., Qian Z. (2026). A multimodal imaging nanobubble enhancing sonodynamic therapy by cell membrane disruption for effective anti-melanoma. Biomaterials.

[149] Wei X., Feng Z., Huang J., Xiang X., Du F., He C., Zhou M., Ma L., Cheng C., Qiu L. (2021). Homology and immune checkpoint dual-targeted sonocatalytic nanoagents for enhancing sonodynamic tumor therapy. ACS Appl. Mater. Interfaces.

[150] Li Y., Chen W., Kang Y., Zhen X., Zhou Z., Liu C., Chen S., Huang X., Liu H.J., Koo S., Kong N., Ji X., Xie T., Tao W. (2023). Nanosensitizer-mediated augmentation of sonodynamic therapy efficacy and antitumor immunity. Nat. Commun..

[151] Huang X., Chen Y., Zhong F., Gui B., Hu Y., Guo Y., Deng Q., Zhou Q. (2024). Targeted ultrasound nanobubbles therapy for prostate cancer via immuno-sonodynamic effect. Int. J. Nanomed..

[152] Nesbitt H., Sheng Y., Kamila S., Logan K., Thomas K., Callan B., Taylor M.A., Love M., O'Rourke D., Kelly P., Beguin E., Stride E., McHale A.P., Callan J.F. (2018). Gemcitabine loaded microbubbles for targeted chemo-sonodynamic therapy of pancreatic cancer. J. Control. Release.

[153] Chen Y., Pang E., Peng R., Tang Y., Tan Q., Lan M., Bai D. (2024). Cationic polythiophene as gene carrier and sonosensitizer for sonodynamic synergic gene therapy of hepatocellular carcinoma. ACS Biomater Sci. Eng..

[154] Li G., Zhang Y., Li J. (2023). A hybrid nanoassembly for ultrasound-inducible cytosolic siRNA delivery and cancer sono-gene therapy. Ultrason. Sonochem..

[155] Price R.J., Skyba D.M., Kaul S., Skalak T.C. (1998). Delivery of colloidal particles and red blood cells to tissue through microvessel ruptures created by targeted microbubble destruction with ultrasound. Circulation.

[156] Miller D.L., Quddus J. (2000). Sonoporation of monolayer cells by diagnostic ultrasound activation of contrast-agent gas bodies. Ultrasound Med. Biol..

[157] Phillips L.C., Klibanov A.L., Wamhoff B.R., Hossack J.A. (2010). Targeted gene transfection from microbubbles into vascular smooth muscle cells using focused, ultrasound-mediated delivery. Ultrasound Med. Biol..

[158] Husseini G.A., Pitt W.G. (2008). Micelles and nanoparticles for ultrasonic drug and gene delivery. Adv. Drug Deliv. Rev..

[159] Tu J., Zhang H., Yu J., Liufu C., Chen Z. (2018). Ultrasound-mediated microbubble destruction: a new method in cancer immunotherapy. Onco Targets Ther.

[160] Paliwal S., Mitragotri S. (2006). Ultrasound-induced cavitation: applications in drug and gene delivery. Expert Opin. Drug Deliv..

[161] Wang T.Y., Choe J.W., Pu K., Devulapally R., Bachawal S., Machtaler S., Chowdhury S.M., Luong R., Tian L., Khuri-Yakub B., Rao J., Paulmurugan R., Willmann J.K. (2015). Ultrasound-guided delivery of microRNA loaded nanoparticles into cancer. J. Control. Release.

[162] Qiu Y., Luo Y., Zhang Y., Cui W., Zhang D., Wu J., Zhang J., Tu J. (2010). The correlation between acoustic cavitation and sonoporation involved in ultrasound-mediated DNA transfection with polyethylenimine (PEI) in vitro. J. Control. Release.

[163] Xu W., Zhang X., Hu X., Zhiyi C., Huang P. (2019). Translational prospects of ultrasound-mediated tumor immunotherapy: Preclinical advances and safety considerations. Cancer Lett..

[164] Li H., Zhang Y., Shu H., Lv W., Su C., Nie F. (2022). Highlights in ultrasound-targeted microbubble destruction-mediated gene/drug delivery strategy for treatment of malignancies. Int. J. Mol. Sci..

[165] Meijering B.D., Juffermans L.J., van Wamel A., Henning R.H., Zuhorn I.S., Emmer M., Versteilen A.M., Paulus W.J., van Gilst W.H., Kooiman K., de Jong N., Musters R.J., Deelman L.E., Kamp O. (2009). Ultrasound and microbubble-targeted delivery of macromolecules is regulated by induction of endocytosis and pore formation. Circ. Res..

[166] Alkins R., Burgess A., Kerbel R., Wels W.S., Hynynen K. (2016). Early treatment of HER2-amplified brain tumors with targeted NK-92 cells and focused ultrasound improves survival. Neuro Oncol..

[167] Downs M.E., Buch A., Sierra C., Karakatsani M.E., Teichert T., Chen S., Konofagou E.E., Ferrera V.P. (2015). Long-term safety of repeated blood-brain barrier opening via focused ultrasound with microbubbles in non-human primates performing a cognitive task. PLoS One.

[168] Zhao Y.Z., Lin Q., Wong H.L., Shen X.T., Yang W., Xu H.L., Mao K.L., Tian F.R., Yang J.J., Xu J., Xiao J., Lu C.T. (2016). Glioma-targeted therapy using Cilengitide nanoparticles combined with UTMD enhanced delivery. J. Control. Release.

[169] Abrahao A., Meng Y., Llinas M., Huang Y., Hamani C., Mainprize T., Aubert I., Heyn C., Black S.E., Hynynen K., Lipsman N., Zinman L. (2019). First-in-human trial of blood-brain barrier opening in amyotrophic lateral sclerosis using MR-guided focused ultrasound. Nat. Commun..

[170] Xing L., Shi Q., Zheng K., Shen M., Ma J., Li F., Liu Y., Lin L., Tu W., Duan Y., Du L. (2016). Ultrasound-mediated microbubble destruction (UMMD) facilitates the delivery of CA19-9 targeted and paclitaxel loaded mPEG-PLGA-PLL nanoparticles in pancreatic cancer. Theranostics.

[171] Sun L., Huang C.W., Wu J., Chen K.J., Li S.H., Weisel R.D., Rakowski H., Sung H.W., Li R.K. (2013). The use of cationic microbubbles to improve ultrasound-targeted gene delivery to the ischemic myocardium. Biomaterials.

[172] Bazan-Peregrino M., Rifai B., Carlisle R.C., Choi J., Arvanitis C.D., Seymour L.W., Coussios C.C. (2013). Cavitation-enhanced delivery of a replicating oncolytic adenovirus to tumors using focused ultrasound. J. Control. Release.

[173] Baetke S.C., Rix A., Tranquart F., Schneider R., Lammers T., Kiessling F., Lederle W. (2016). Squamous cell carcinoma xenografts: use of VEGFR2-targeted microbubbles for combined functional and molecular US to monitor antiangiogenic therapy effects. Radiology.

[174] Oishi Y., Kakimoto T., Yuan W., Kuno S., Yamashita H., Chiba T. (2016). Fetal gene therapy for ornithine transcarbamylase deficiency by intrahepatic plasmid DNA-Micro-bubble injection combined with hepatic ultrasound insonation. Ultrasound Med. Biol..

[175] Liu Y., Jiang J., Liu C., Zhao W., Ma Y., Zheng Z., Zhou Q., Zhao Y. (2021). Synergistic anti-tumor effect of anti-PD-L1 antibody cationic microbubbles for delivery of the miR-34a gene combined with ultrasound on cervical carcinoma. Am. J. Transl. Res..

[176] Chen J., Qiu S., Liu Y., Sun W., Zhou T., Zhao L., Li Z., Duan Y. (2024). Ultrasound targeted microbubble destruction assisted exosomal delivery of siHmox1 effectively inhibits doxorubicin-induced cardiomyocyte ferroptosis. J. Nanobiotechnol..

[177] Wang S., Chen K., Wang Y., Wang Z., Li Z., Guo J., Chen J., Liu W., Guo X., Yan G., Liang C., Yu H., Fang S., Yu B. (2023). Cardiac-targeted delivery of nuclear receptor RORα via ultrasound targeted microbubble destruction optimizes the benefits of regular dose of melatonin on sepsis-induced cardiomyopathy. Biomater. Res..

[178] Bray F., Laversanne M., Sung H., Ferlay J., Siegel R.L., Soerjomataram I., Jemal A. (2024). Global cancer statistics2022: GLOBOCAN estimates of incidence and mortality worldwide for 36 cancers in 185 countries. CA Cancer J. Clin..

[179] Najafi M., Goradel N.H., Farhood B., Salehi E., Solhjoo S., Toolee H., Kharazinejad E., Mortezaee K. (2019). Tumor microenvironment: Interactions and therapy. J. Cell. Physiol..

[180] Binnewies M., Roberts E.W., Kersten K., Chan V., Fearon D.F., Merad M., Coussens L.M., Gabrilovich D.I., Ostrand-Rosenberg S., Hedrick C.C., Vonderheide R.H., Pittet M.J., Jain R.K., Zou W., Howcroft T.K., Woodhouse E.C., Weinberg R.A., Krummel M.F. (2018). Understanding the tumor immune microenvironment (TIME) for effective therapy. Nat. Med..

[181] Brown J.M., Giaccia A.J. (1998). The unique physiology of solid tumors: opportunities (and problems) for cancer therapy. Cancer Res..

[182] Noman M.Z., Hasmim M., Lequeux A., Xiao M., Duhem C., Chouaib S., Berchem G., Janji B. (2019). Improving cancer immunotherapy by targeting the hypoxic tumor microenvironment: new opportunities and challenges. Cells.

[183] Meacham C.E., Morrison S.J. (2013). Tumour heterogeneity and cancer cell plasticity. Nature.

[184] Debela D.T., Muzazu S.G., Heraro K.D., Ndalama M.T., Mesele B.W., Haile D.C., Kitui S.K., Manyazewal T. (2021). New approaches and procedures for cancer treatment: current perspectives. SAGE Open Med.

[185] Anderson K.G., Stromnes I.M., Greenberg P.D. (2017). Obstacles posed by the tumor microenvironment to T cell activity: a case for synergistic therapies. Cancer Cell.

[186] Stylianopoulos T., Munn L.L., Jain R.K. (2018). Reengineering the physical microenvironment of tumors to improve drug delivery and efficacy: from mathematical modeling to bench to bedside. Trends Cancer.

[187] ter Haar G., Sinnett D., Rivens I. (1989). High intensity focused ultrasound–a surgical technique for the treatment of discrete liver tumours. Phys. Med. Biol..

[188] Hynynen K., Jolesz F.A. (1998). Demonstration of potential noninvasive ultrasound brain therapy through an intact skull. Ultrasound Med. Biol..

[189] Miller D.L., Smith N.B., Bailey M.R., Czarnota G.J., Hynynen K., Makin I.R. (2012). Overview of therapeutic ultrasound applications and safety considerations. J. Ultrasound Med..

[190] Thim E.A., Kitelinger L.E., Rivera-Escalera F., Mathew A.S., Elliott M.R., Bullock T.N.J., Price R.J. (2024). Focused ultrasound ablation of melanoma with boiling histotripsy yields abscopal tumor control and antigen-dependent dendritic cell activation. Theranostics.

[191] Zhao H., Du F., Xiang X., Tang Y., Feng Z., Wang Z., Rong X., Qiu L. (2024). Progress in application of nanomedicines for enhancing cancer sono-immunotherapy. Ultrason. Sonochem..

[192] Liu B., Du F., Feng Z., Xiang X., Guo R., Ma L., Zhu B., Qiu L. (2024). Ultrasound-augmented cancer immunotherapy. J. Mater. Chem. B.

[193] Xiang X., Pang H., Ma T., Du F., Li L., Huang J., Ma L., Qiu L. (2021). Ultrasound targeted microbubble destruction combined with Fe-MOF based bio-/enzyme-mimics nanoparticles for treating of cancer. J. Nanobiotechnol..

[194] Zhang Q., Jin H., Chen L., Chen Q., He Y., Yang Y., Ma S., Xiao S., Xi F., Luo Q., Liu J. (2019). Effect of ultrasound combined with microbubble therapy on interstitial fluid pressure and VX2 tumor structure in rabbit. Front. Pharmacol..

[195] Do H.D., Marie C., Bessoles S., Dhotel H., Seguin J., Larrat B., Doan B.T., Scherman D., Escriou V., Hacein-Bey-Abina S., Mignet N. (2022). Combination of thermal ablation by focused ultrasound, pFAR4-IL-12 transfection and lipidic adjuvant provide a distal immune response. Explor. Targeted Anti-Tumor Ther..

[196] van den Bijgaart R.J., Eikelenboom D.C., Hoogenboom M., Fütterer J.J., den Brok M.H., Adema G.J. (2017). Thermal and mechanical high-intensity focused ultrasound: perspectives on tumor ablation, immune effects and combination strategies. Cancer Immunol Immunother.

[197] Abe S., Nagata H., Crosby E.J., Inoue Y., Kaneko K., Liu C.X., Yang X., Wang T., Acharya C.R., Agarwal P., Snyder J., Gwin W., Morse M.A., Zhong P., Lyerly H.K., Osada T. (2022). Combination of ultrasound-based mechanical disruption of tumor with immune checkpoint blockade modifies tumor microenvironment and augments systemic antitumor immunity. J. Immunother. Cancer.

[198] Zhao W.P., Chen J.Y., Zhang L., Li Q., Qin J., Peng S., Li K.Q., Wang Z.B., Chen W.Z. (2013). Feasibility of ultrasound-guided high intensity focused ultrasound ablating uterine fibroids with hyperintense on T2-weighted MR imaging. Eur. J. Radiol..

[199] Yang Q., Nanayakkara G.K., Drummer C., Sun Y., Johnson C., Cueto R., Fu H., Shao Y., Wang L., Yang W.Y., Tang P., Liu L.W., Ge S., Zhou X.D., Khan M., Wang H., Yang X. (2017). Low-intensity ultrasound-induced anti-inflammatory effects are mediated by several new mechanisms including gene induction, immunosuppressor cell promotion, and enhancement of exosome biogenesis and docking. Front. Physiol..

[200] Li M., Wan G., Yu H., Xiong W. (2019). High-intensity focused ultrasound inhibits invasion and metastasis of colon cancer cells by enhancing microRNA-124-mediated suppression of STAT3. FEBS Open Bio.

[201] Dewitte H., Van Lint S., Heirman C., Thielemans K., De Smedt S.C., Breckpot K., Lentacker I. (2014). The potential of antigen and TriMix sonoporation using mRNA-loaded microbubbles for ultrasound-triggered cancer immunotherapy. J. Control. Release.

[202] Suzuki R., Namai E., Oda Y., Nishiie N., Otake S., Koshima R., Hirata K., Taira Y., Utoguchi N., Negishi Y., Nakagawa S., Maruyama K. (2010). Cancer gene therapy by IL-12 gene delivery using liposomal bubbles and tumoral ultrasound exposure. J. Control. Release.

[203] Chowdhury S.M., Lee T., Bachawal S.V., Devulapally R., Abou-Elkacem L., Yeung T.A., Wischhusen J., Tian L., Dahl J., Paulmurugan R., Willmann J.K. (2018). Longitudinal assessment of ultrasound-guided complementary microRNA therapy of hepatocellular carcinoma. J. Control. Release.

[204] Anastasiadis P., Gandhi D., Guo Y., Ahmed A.K., Bentzen S.M., Arvanitis C., Woodworth G.F. (2021). Localized blood-brain barrier opening in infiltrating gliomas with MRI-guided acoustic emissions-controlled focused ultrasound. PNAS.

[205] Meng Y., Reilly R.M., Pezo R.C., Trudeau M., Sahgal A., Singnurkar A., Perry J., Myrehaug S., Pople C.B., Davidson B., Llinas M., Hyen C., Huang Y., Hamani C., Suppiah S., Hynynen K., Lipsman N. (2021). MR-guided focused ultrasound enhances delivery of trastuzumab to Her2-positive brain metastases. Sci. Transl. Med..

[206] Guo Y., Lee H., Fang Z., Velalopoulou A., Kim J., Thomas M.B., Liu J., Abramowitz R.G., Kim Y., Coskun A.F., Krummel D.P., Sengupta S., MacDonald T.J., Arvanitis C. (2021). Single-cell analysis reveals effective siRNA delivery in brain tumors with microbubble-enhanced ultrasound and cationic nanoparticles. Sci. Adv..

[207] Yang Q., Zhou Y., Chen J., Huang N., Wang Z., Cheng Y. (2021). Gene therapy for drug-resistant glioblastoma via lipid-polymer hybrid nanoparticles combined with focused ultrasound. Int. J. Nanomed..

[208] Chen Y., Du M., Yuan Z., Chen Z., Yan F. (2022). Spatiotemporal control of engineered bacteria to express interferon-γ by focused ultrasound for tumor immunotherapy. Nat. Commun..

[209] Han H., Kim D., Jang Y., Seo M., Kim K., Lee J.B., Kim H. (2020). Focused ultrasound-triggered chemo-gene therapy with multifunctional nanocomplex for enhancing therapeutic efficacy. J. Control. Release.

[210] de Teresa E. (1997). Beta-blockers in cardiac insufficiency: should they always be considered in the therapeutic strategy? Arguments in favor. Rev. Esp. Cardiol..

[211] Tomaniak M., Katagiri Y., Modolo R., de Silva R., Khamis R.Y., Bourantas C.V., Torii R., Wentzel J.J., Gijsen F.J.H., van Soest G., Stone P.H., West N.E.J., Maehara A., Lerman A., van der Steen A.F.W., Lüscher T.F., Virmani R., Koenig W., Stone G.W., Muller J.E., Wijns W., Serruys P.W., Onuma Y. (2020). Vulnerable plaques and patients: state-of-the-art. Eur. Heart J..

[212] Virmani R., Burke A.P., Farb A., Kolodgie F.D. (2006). Pathology of the vulnerable plaque. J. Am. Coll. Cardiol..

[213] Datta S., Coussios C.C., McAdory L.E., Tan J., Porter T., De Courten-Myers G., Holland C.K. (2006). Correlation of cavitation with ultrasound enhancement of thrombolysis. Ultrasound Med. Biol..

[214] Yu G.Z., Istvanic F., Chen X., Nouraie M., Shiva S., Straub A.C., Pacella J.J. (2020). Ultrasound-targeted microbubble cavitation with sodium nitrite synergistically enhances nitric oxide production and microvascular perfusion. Ultrasound Med. Biol..

[215] Heo J., Park J.H., Kim H.J., Pahk K., Pahk K.J. (2023). Sonothrombolysis with an acoustic net-assisted boiling histotripsy: a proof-of-concept study. Ultrason. Sonochem..

[216] Rosenschein U., Furman V., Kerner E., Fabian I., Bernheim J., Eshel Y. (2000). Ultrasound imaging-guided noninvasive ultrasound thrombolysis: preclinical results. Circulation.

[217] Xie F., Lof J., Everbach C., He A., Bennett R.M., Matsunaga T., Johanning J., Porter T.R. (2009). Treatment of acute intravascular thrombi with diagnostic ultrasound and intravenous microbubbles, JACC: Cardiovasc. Imaging.

[218] Mathias W., Tsutsui J.M., Tavares B.G., Xie F., Aguiar M.O., Garcia D.R., Oliveira M.T., Soeiro A., Nicolau J.C., Lemos P.A.N., Rochitte C.E., Ramires J.A., Kalil R.F., Porter T.R. (2016). Diagnostic ultrasound impulses improve microvascular flow in patients with STEMI receiving intravenous microbubbles. J. Am. Coll. Cardiol..

[219] Xie F., Gao S., Wu J., Lof J., Radio S., Vignon F., Shi W., Powers J., Unger E., Everbach E.C., Liu J., Porter T.R. (2013). Diagnostic ultrasound induced inertial cavitation to non-invasively restore coronary and microvascular flow in acute myocardial infarction. PLoS One.

[220] Mathias W., Tsutsui J.M., Tavares B.G., Fava A.M., Aguiar M.O.D., Borges B.C., Oliveira M.T., Soeiro A., Nicolau J.C., Ribeiro H.B., Chiang H.P., Sbano J.C.N., Morad A., Goldsweig A., Rochitte C.E., Lopes B.B.C., Ramirez J.A.F., Kalil Filho R., Porter T.R. (2019). Sonothrombolysis in ST-segment elevation myocardial infarction treated with primary percutaneous coronary intervention. J. Am. Coll. Cardiol..

[221] Aguiar M.O.D., Tavares B.G., Tsutsui J.M., Fava A.M., Borges B.C., Oliveira M.T., Soeiro A., Nicolau J.C., Ribeiro H.B., Chiang H.P., Sbano J.C.N., Goldsweig A., Rochitte C.E., Lopes B.B.C., Ramirez J.A.F., Kalil Filho R., Porter T.R., Mathias W. (2020). Sonothrombolysis improves myocardial dynamics and microvascular obstruction preventing left ventricular remodeling in patients with ST elevation myocardial infarction. Circ. Cardiovasc. Imaging.

[222] Nishida T., Shimokawa H., Oi K., Tatewaki H., Uwatoku T., Abe K., Matsumoto Y., Kajihara N., Eto M., Matsuda T., Yasui H., Takeshita A., Sunagawa K. (2004). Extracorporeal cardiac shock wave therapy markedly ameliorates ischemia-induced myocardial dysfunction in pigs in vivo. Circulation.

[223] Kikuchi Y., Ito K., Ito Y., Shiroto T., Tsuburaya R., Aizawa K., Hao K., Fukumoto Y., Takahashi J., Takeda M., Nakayama M., Yasuda S., Kuriyama S., Tsuji I., Shimokawa H. (2010). Double-blind and placebo-controlled study of the effectiveness and safety of extracorporeal cardiac shock wave therapy for severe angina pectoris. Circ J.

[224] Shimokawa H., Miura M., Nochioka K., Sakata Y. (2015). Heart failure as a general pandemic in Asia. Eur. J. Heart Fail..

[225] Owan T.E., Hodge D.O., Herges R.M., Jacobsen S.J., Roger V.L., Redfield M.M. (2006). Trends in prevalence and outcome of heart failure with preserved ejection fraction. N. Engl. J. Med..

[226] Roger V.L. (2021). Epidemiology of heart failure: a contemporary perspective. Circ. Res..

[227] Lourenço A.P., Leite-Moreira A.F., Balligand J.L., Bauersachs J., Dawson D., de Boer R.A., de Windt L.J., Falcão-Pires I., Fontes-Carvalho R., Franz S., Giacca M., Hilfiker-Kleiner D., Hirsch E., Maack C., Mayr M., Pieske B., Thum T., Tocchetti C.G., Brutsaert D.L., Heymans S. (2018). An integrative translational approach to study heart failure with preserved ejection fraction: a position paper from the Working Group on Myocardial Function of the European Society of Cardiology. Eur. J. Heart Fail..

[228] Redfield M.M., Jacobsen S.J., Burnett J.C., Mahoney D.W., Bailey K.R., Rodeheffer R.J. (2003). Burden of systolic and diastolic ventricular dysfunction in the community: appreciating the scope of the heart failure epidemic. J. Am. Med. Assoc..

[229] Shindo T., Ito K., Ogata T., Hatanaka K., Kurosawa R., Eguchi K., Kagaya Y., Hanawa K., Aizawa K., Shiroto T., Kasukabe S., Miyata S., Taki H., Hasegawa H., Kanai H., Shimokawa H. (2016). Low-intensity pulsed ultrasound enhances angiogenesis and ameliorates left ventricular dysfunction in a mouse model of acute myocardial infarction. Arterioscler. Thromb. Vasc. Biol..

[230] Nakata T., Shindo T., Ito K., Eguchi K., Monma Y., Ichijo S., Ryoke R., Satoh W., Kumasaka K., Sato H., Kurosawa R., Satoh K., Kawashima R., Miura M., Kanai H., Yasuda S., Shimokawa H. (2023). Beneficial effects of low-intensity pulsed ultrasound therapy on right ventricular dysfunction in animal models. JACC Basic Transl Sci.

[231] Monma Y., Shindo T., Eguchi K., Kurosawa R., Kagaya Y., Ikumi Y., Ichijo S., Nakata T., Miyata S., Matsumoto A., Sato H., Miura M., Kanai H., Shimokawa H. (2021). Low-intensity pulsed ultrasound ameliorates cardiac diastolic dysfunction in mice: a possible novel therapy for heart failure with preserved left ventricular ejection fraction. Cardiovasc. Res..

[232] Feigin V.L., Lawes C.M., Bennett D.A., Barker-Collo S.L., Parag V. (2009). Worldwide stroke incidence and early case fatality reported in 56 population-based studies: a systematic review. Lancet Neurol..

[233] Hayakawa K., Esposito E., Wang X., Terasaki Y., Liu Y., Xing C., Ji X., Lo E.H. (2016). Transfer of mitochondria from astrocytes to neurons after stroke. Nature.

[234] Saqqur M., Tsivgoulis G., Nicoli F., Skoloudik D., Sharma V.K., Larrue V., Eggers J., Perren F., Charalampidis P., Storie D., Shuaib A., Alexandrov A.V. (2014). The role of sonolysis and sonothrombolysis in acute ischemic stroke: a systematic review and meta-analysis of randomized controlled trials and case-control studies. J. Neuroimaging.

[235] Barreto A.D., Sharma V.K., Lao A.Y., Schellinger P.D., Amarenco P., Sierzenski P., Alexandrov A.V., Molina C.A. (2009). Safety and dose-escalation study design of transcranial ultrasound in clinical SONolysis for acute ischemic stroke: the TUCSON trial. Int. J. Stroke.

[236] Barreto A.D., Alexandrov A.V., Shen L., Sisson A., Bursaw A.W., Sahota P., Peng H., Ardjomand-Hessabi M., Pandurengan R., Rahbar M.H., Barlinn K., Indupuru H., Gonzales N.R., Savitz S.I., Grotta J.C. (2013). CLOTBUST-Hands Free: pilot safety study of a novel operator-independent ultrasound device in patients with acute ischemic stroke. Stroke.

[237] Daffertshofer M., Gass A., Ringleb P., Sitzer M., Sliwka U., Els T., Sedlaczek O., Koroshetz W.J., Hennerici M.G. (2005). Transcranial low-frequency ultrasound-mediated thrombolysis in brain ischemia: increased risk of hemorrhage with combined ultrasound and tissue plasminogen activator: results of a phase II clinical trial. Stroke.

[238] Choi W., Key J., Youn I., Lee H., Han S. (2022). Cavitation-assisted sonothrombolysis by asymmetrical nanostars for accelerated thrombolysis. J. Control. Release.

[239] Li Y., Teng X., Yang C., Wang Y., Wang L., Dai Y., Sun H., Li J. (2021). Ultrasound controlled anti-inflammatory polarization of platelet decorated microglia for targeted ischemic stroke therapy. Angew. Chem. Int. Ed..

[240] Lin C.Y., Tsai C.H., Feng L.Y., Chai W.Y., Lin C.J., Huang C.Y., Wei K.C., Yeh C.K., Chen C.M., Liu H.L. (2019). Focused ultrasound-induced blood brain-barrier opening enhanced vascular permeability for GDNF delivery in Huntington's disease mouse model. Brain Stimul..

[241] Picillo M., Fasano A. (2016). Recent advances in essential tremor: surgical treatment. Parkinsonism Relat. Disord..

[242] Pardridge W.M. (2020). Treatment of Alzheimer's disease and blood-brain barrier drug delivery. Pharmaceuticals (Basel).

[243] Lipsman N., Meng Y., Bethune A.J., Huang Y., Lam B., Masellis M., Herrmann N., Heyn C., Aubert I., Boutet A., Smith G.S., Hynynen K., Black S.E. (2018). Blood-brain barrier opening in Alzheimer's disease using MR-guided focused ultrasound. Nat. Commun..

[244] Gasca-Salas C., Fernández-Rodríguez B., Pineda-Pardo J.A., Rodríguez-Rojas R., Obeso I., Hernández-Fernández F., Del Álamo M., Mata D., Guida P., Ordás-Bandera C., Montero-Roblas J.I., Martínez-Fernández R., Foffani G., Rachmilevitch I., Obeso J.A. (2021). Blood-brain barrier opening with focused ultrasound in Parkinson's disease dementia. Nat. Commun..

[245] Mainprize T., Lipsman N., Huang Y., Meng Y., Bethune A., Ironside S., Heyn C., Alkins R., Trudeau M., Sahgal A., Perry J., Hynynen K. (2019). Blood-brain barrier opening in primary brain tumors with non-invasive MR-guided focused ultrasound: a clinical safety and feasibility study. Sci. Rep..

[246] Krishna V., Sammartino F., Rezai A. (2018). A review of the current therapies, challenges, and future directions of transcranial focused ultrasound technology: advances in diagnosis and treatment. JAMA Neurol..

[247] Rezai A.R., Ranjan M., D'Haese P.F., Haut M.W., Carpenter J., Najib U., Mehta R.I., Chazen J.L., Zibly Z., Yates J.R., Hodder S.L., Kaplitt M. (2020). Noninvasive hippocampal blood-brain barrier opening in Alzheimer's disease with focused ultrasound. PNAS.

[248] Park S.H., Baik K., Jeon S., Chang W.S., Ye B.S., Chang J.W. (2021). Extensive frontal focused ultrasound mediated blood-brain barrier opening for the treatment of Alzheimer's disease: a proof-of-concept study. Transl. Neurodegener..

[249] Rezai A.R., Ranjan M., Haut M.W., Carpenter J., D'Haese P.F., Mehta R.I., Najib U., Wang P., Claassen D.O., Chazen J.L., Krishna V., Deib G., Zibly Z., Hodder S.L., Wilhelmsen K.C., Finomore V., Konrad P.E., Kaplitt M. (2023). Focused ultrasound-mediated blood-brain barrier opening in Alzheimer's disease: long-term safety, imaging, and cognitive outcomes. J. Neurosurg..

[250] Rezai A.R., D'Haese P.F., Finomore V., Carpenter J., Ranjan M., Wilhelmsen K., Mehta R.I., Wang P., Najib U., Vieira Ligo Teixeira C., Arsiwala T., Tarabishy A., Tirumalai P., Claassen D.O., Hodder S., Haut M.W. (2024). Ultrasound blood-brain barrier opening and aducanumab in Alzheimer's disease. N. Engl. J. Med..

[251] Borovikova L.V., Ivanova S., Zhang M., Yang H., Botchkina G.I., Watkins L.R., Wang H., Abumrad N., Eaton J.W., Tracey K.J. (2000). Vagus nerve stimulation attenuates the systemic inflammatory response to endotoxin. Nature.

[252] Bai A., Guo Y., Lu N. (2007). The effect of the cholinergic anti-inflammatory pathway on experimental colitis. Scand. J. Immunol..

[253] Zachs D.P., Offutt S.J., Graham R.S., Kim Y., Mueller J., Auger J.L., Schuldt N.J., Kaiser C.R.W., Heiller A.P., Dutta R., Guo H., Alford J.K., Binstadt B.A., Lim H.H. (2019). Noninvasive ultrasound stimulation of the spleen to treat inflammatory arthritis. Nat. Commun..

[254] Lin J., Zhang W., Jones A., Doherty M. (2004). Efficacy of topical non-steroidal anti-inflammatory drugs in the treatment of osteoarthritis: meta-analysis of randomised controlled trials. BMJ.

[255] Vinikoor T., Dzidotor G.K., Le T.T., Liu Y., Kan H.M., Barui S., Chorsi M.T., Curry E.J., Reinhardt E., Wang H., Singh P., Merriman M.A., D'Orio E., Park J., Xiao S., Chapman J.H., Lin F., Truong C.S., Prasadh S., Chuba L., Killoh S., Lee S.W., Wu Q., Chidambaram R.M., Lo K.W.H., Laurencin C.T., Nguyen T.D. (2023). Injectable and biodegradable piezoelectric hydrogel for osteoarthritis treatment. Nat. Commun..

[256] Johnson L., Igoe E., Kleftouris G., Papachristos I.V., Papakostidis C., Giannoudis P.V. (2019). Physical health and psychological outcomes in adult patients with long-bone fracture non-unions: evidence today. J. Clin. Med..

[257] Li Z., Liu J., Li C., Wu M., Li Y., Cui Y., Xiong W., Yang F., Liu B. (2023). Advances in the application of bone transport techniques in the treatment of bone nonunion and bone defects. Orthop. Surg..

[258] Hu S., Wang S., He Q., Li D., Xin L., Xu C., Zhu X., Mei L., Cannon R.D., Ji P., Tang H., Chen T. (2023). A mechanically reinforced super bone glue makes a leap in hard tissue strong adhesion and augmented bone regeneration. Adv. Sci. (Weinh).

[259] Laubach M., Suresh S., Herath B., Wille M.L., Delbrück H., Alabdulrahman H., Hutmacher D.W., Hildebrand F. (2022). Clinical translation of a patient-specific scaffold-guided bone regeneration concept in four cases with large long bone defects. J. Orthop. Translat..

[260] Shen M.J., Wang C.Y., Hao D.X., Hao J.X., Zhu Y.F., Han X.X., Tonggu L., Chen J.H., Jiao K., Tay F.R., Niu L.N. (2022). Multifunctional nanomachinery for enhancement of bone healing. Adv. Mater..

[261] Zhang D., Zheng H., Geng K., Shen J., Feng X., Xu P., Duan Y., Li Y., Wu R., Gou Z., Gao C. (2021). Large fuzzy biodegradable polyester microspheres with dopamine deposition enhance cell adhesion and bone regeneration in vivo. Biomaterials.

[262] Yu T., Zhang L., Dou X., Bai R., Wang H., Deng J., Zhang Y., Sun Q., Li Q., Wang X., Han B. (2022). Mechanically robust hydrogels facilitating bone regeneration through epigenetic modulation. Adv. Sci. (Weinh).

[263] da Silva L.P., Kundu S.C., Reis R.L., Correlo V.M. (2020). Electric phenomenon: a disregarded tool in tissue engineering and regenerative medicine. Trends Biotechnol..

[264] Khalifeh J.M., Zohny Z., MacEwan M., Stephen M., Johnston W., Gamble P., Zeng Y., Yan Y., Ray W.Z. (2018). Electrical stimulation and bone healing: a review of current technology and clinical applications. IEEE Rev. Biomed. Eng..

[265] Takizawa T., Nakayama N., Haniu H., Aoki K., Okamoto M., Nomura H., Tanaka M., Sobajima A., Yoshida K., Kamanaka T., Ajima K., Oishi A., Kuroda C., Ishida H., Okano S., Kobayashi S., Kato H., Saito N. (2018). Titanium fiber plates for bone tissue repair. Adv. Mater..

[266] Silva D., Guerra C., Muñoz H., Aguilar C., Walter M., Azocar M., Muñoz L., Gürbüz E., Ringuedé A., Cassir M., Sancy M. (2020). The effect of Staphylococcus aureus on the electrochemical behavior of porous Ti-6Al-4V alloy. Bioelectrochemistry.

[267] Rajabi A.H., Jaffe M., Arinzeh T.L. (2015). Piezoelectric materials for tissue regeneration: a review. Acta Biomater..

[268] Park J.B., Kelly B.J., Kenner G.H., von Recum A.F., Grether M.F., Coffeen W.W. (1981). Piezoelectric ceramic implants: in vivo results. J. Biomed. Mater. Res..

[269] Dubey A.K., Thrivikraman G., Basu B. (2015). Absence of systemic toxicity in mouse model towards BaTiO3 nanoparticulate based eluate treatment. J. Mater. Sci. - Mater. Med..

[270] Zhou S., Xiao C., Fan L., Yang J., Ge R., Cai M., Yuan K., Li C., Crawford R.W., Xiao Y., Yu P., Deng C., Ning C., Zhou L., Wang Y. (2024). Injectable ultrasound-powered bone-adhesive nanocomposite hydrogel for electrically accelerated irregular bone defect healing. J. Nanobiotechnol..

[271] Bez M., Sheyn D., Tawackoli W., Avalos P., Shapiro G., Giaconi J.C., Da X., David S.B., Gavrity J., Awad H.A., Bae H.W., Ley E.J., Kremen T.J., Gazit Z., Ferrara K.W., Pelled G., Gazit D. (2017). In situ bone tissue engineering via ultrasound-mediated gene delivery to endogenous progenitor cells in mini-pigs. Sci. Transl. Med..

[272] Liu J., Saul D., Böker K.O., Ernst J., Lehman W., Schilling A.F. (2018). Current methods for skeletal muscle tissue repair and regeneration. Biomed Res. Int..

[273] Moyer A.L., Wagner K.R. (2011). Regeneration versus fibrosis in skeletal muscle. Curr. Opin. Rheumatol..

[274] Tidball J.G. (2017). Regulation of muscle growth and regeneration by the immune system. Nat. Rev. Immunol..

[275] Nahrendorf M., Swirski F.K. (2016). Abandoning M1/M2 for a network model of macrophage function. Circ. Res..

[276] Shapouri-Moghaddam A., Mohammadian S., Vazini H., Taghadosi M., Esmaeili S.A., Mardani F., Seifi B., Mohammadi A., Afshari J.T., Sahebkar A. (2018). Macrophage plasticity, polarization, and function in health and disease. J. Cell. Physiol..

[277] Luo Z.W., Sun Y.Y., Lin J.R., Qi B.J., Chen J.W. (2021). Exosomes derived from inflammatory myoblasts promote M1 polarization and break the balance of myoblast proliferation/differentiation, World. J. Stem Cells.

[278] Perandini L.A., Chimin P., Lutkemeyer D.D.S., Câmara N.O.S. (2018). Chronic inflammation in skeletal muscle impairs satellite cells function during regeneration: can physical exercise restore the satellite cell niche?. FEBS J..

[279] Luo Z., Lin J., Sun Y., Wang C., Chen J. (2021). Bone marrow stromal cell-derived exosomes promote muscle healing following contusion through macrophage polarization. Stem Cells Dev..

[280] Qin H., Luo Z., Sun Y., He Z., Qi B., Chen Y., Wang J., Li C., Lin W., Han Z., Zhu Y. (2023). Low-intensity pulsed ultrasound promotes skeletal muscle regeneration via modulating the inflammatory immune microenvironment. Int. J. Biol. Sci..

[281] Chan Y.S., Hsu K.Y., Kuo C.H., Lee S.D., Chen S.C., Chen W.J., Ueng S.W. (2010). Using low-intensity pulsed ultrasound to improve muscle healing after laceration injury: an in vitro and in vivo study. Ultrasound Med. Biol..

[282] Hu J., Qu J., Xu D., Zhang T., Qin L., Lu H. (2014). Combined application of low-intensity pulsed ultrasound and functional electrical stimulation accelerates bone-tendon junction healing in a rabbit model. J. Orthop. Res..

[283] Montalti C.S., Souza N.V., Rodrigues N.C., Fernandes K.R., Toma R.L., Renno A.C. (2013). Effects of low-intensity pulsed ultrasound on injured skeletal muscle. Braz. J. Phys. Ther..

[284] Chen J., Jiang J., Wang W., Qin J., Chen J., Chen W., Wang Y. (2019). Low intensity pulsed ultrasound promotes the migration of bone marrow- derived mesenchymal stem cells via activating FAK-ERK1/2 signalling pathway, Artif Cells Nanomed. Biotechnol.

[285] Ling L., Wei T., He L., Wang Y., Wang Y., Feng X., Zhang W., Xiong Z. (2017). Low-intensity pulsed ultrasound activates ERK1/2 and PI3K-akt signalling pathways and promotes the proliferation of human amnion-derived mesenchymal stem cells. Cell Prolif..

[286] He R., Zhou W., Zhang Y., Hu S., Yu H., Luo Y., Liu B., Ran J., Wu J., Wang Y., Chen W. (2015). Combination of low-intensity pulsed ultrasound and C3H10T1/2 cells promotes bone-defect healing. Int. Orthop..

[287] He R., Chen J., Jiang J., Liu B., Liang D., Zhou W., Chen W., Wang Y. (2019). Synergies of accelerating differentiation of bone marrow mesenchymal stem cells induced by low intensity pulsed ultrasound, osteogenic and endothelial inductive agent, Artif Cells Nanomed. Biotechnol.

[288] Ghanem B., Seoane-Vazquez E., Brown L., Rodriguez-Monguio R. (2023). Analysis of the Gene Therapies Authorized by the United States Food and Drug Administration and the European Medicines Agency. Med. Care.

[289] Ginn S.L., Amaya A.K., Alexander I.E., Edelstein M., Abedi M.R. (2018). Gene therapy clinical trials worldwide to 2017: an update. J. Gene Med..

[290] Nauber R., Goudu S.R., Goeckenjan M., Bornhäuser M., Ribeiro C., Medina-Sánchez M. (2023). Medical microrobots in reproductive medicine from the bench to the clinic. Nat. Commun..

[291] Huang C., Zhang H., Bai R. (2017). Advances in ultrasound-targeted microbubble-mediated gene therapy for liver fibrosis. Acta Pharm. Sin. B.

[292] Jarow J.P., Baxley J.H. (2015). Medical devices: US medical device regulation. Urol. Oncol..

[293] Darrow J.J., Avorn J., Kesselheim A.S. (2021). FDA Regulation and Approval of Medical Devices: 1976-2020. J. Am. Med. Assoc..

[294] Lauritsen K.J., Nguyen T. (2009). Combination products regulation at the FDA. Clin. Pharmacol. Ther..

[295] Reis M.E., Bettencourt A., Ribeiro H.M. (2022). The regulatory challenges of innovative customized combination products. Front. Med. (Lausanne).

[296] Chen J., Ratnayaka S., Alford A., Kozlovskaya V., Liu F., Xue B., Hoyt K., Kharlampieva E. (2017). Theranostic multilayer capsules for ultrasound imaging and guided drug delivery. ACS Nano.

[297] Misra S.K., Ghoshal G., Gartia M.R., Wu Z., De A.K., Ye M., Bromfield C.R., Williams E.M., Singh K., Tangella K.V., Rund L., Schulten K., Schook L.B., Ray P.S., Burdette E.C., Pan D. (2015). Trimodal therapy: combining hyperthermia with repurposed bexarotene and ultrasound for treating liver cancer. ACS Nano.

[298] Ektate K., Kapoor A., Maples D., Tuysuzoglu A., VanOsdol J., Ramasami S., Ranjan A. (2016). Motion compensated ultrasound imaging allows thermometry and image guided drug delivery monitoring from echogenic liposomes. Theranostics.

[299] Medina S.H., Michie M.S., Miller S.E., Schnermann M.J., Schneider J.P. (2017). Fluorous phase-directed peptide assembly affords nano-peptisomes capable of ultrasound-triggered cellular delivery. Angew. Chem. Int. Ed..

[300] Lin Y.J., Huang C.C., Wan W.L., Chiang C.H., Chang Y., Sung H.W. (2017). Recent advances in CO(2) bubble-generating carrier systems for localized controlled release. Biomaterials.

[301] Wang X., Chen H., Zhang K., Ma M., Li F., Zeng D., Zheng S., Chen Y., Jiang L., Xu H., Shi J. (2014). An intelligent nanotheranostic agent for targeting, redox-responsive ultrasound imaging, and imaging-guided high-intensity focused ultrasound synergistic therapy. Small.

[302] Nam K., Stanczak M., Forsberg F., Liu J.B., Eisenbrey J.R., Solomides C.C., Lyshchik A. (2018). Sentinel lymph node characterization with a dual-targeted molecular ultrasound contrast agent. Mol. Imaging Biol..

[303] Liu H., Chen Y., Yan F., Han X., Wu J., Liu X., Zheng H. (2015). Ultrasound molecular imaging of vascular endothelial growth factor receptor 2 expression for endometrial receptivity evaluation. Theranostics.

[304] Yang F., Li M., Liu Y., Wang T., Feng Z., Cui H., Gu N. (2016). Glucose and magnetic-responsive approach toward in situ nitric oxide bubbles controlled generation for hyperglycemia theranostics. J. Control. Release.

[305] Mullick Chowdhury S., Lee T., Willmann J.K. (2017). Ultrasound-guided drug delivery in cancer. Ultrasonography.

[306] Hynynen K., McDannold N., Vykhodtseva N., Jolesz F.A. (2001). Noninvasive MR imaging-guided focal opening of the blood-brain barrier in rabbits. Radiology.

[307] Fan C.H., Liu H.L., Huang C.Y., Ma Y.J., Yen T.C., Yeh C.K. (2012). Detection of intracerebral hemorrhage and transient blood-supply shortage in focused-ultrasound-induced blood-brain barrier disruption by ultrasound imaging. Ultrasound Med. Biol..

[308] Weng J.C., Wu S.K., Yang F.Y., Lin W.L., Tseng W.Y. (2010). Pulse sequence and timing of contrast-enhanced MRI for assessing blood-brain barrier disruption after transcranial focused ultrasound in the presence of hemorrhage. J. Magn. Reson..

[309] Jones C.H., Chen C.K., Ravikrishnan A., Rane S., Pfeifer B.A. (2013). Overcoming nonviral gene delivery barriers: perspective and future. Mol. Pharm..

[310] Li Y.S., Davidson E., Reid C.N., McHale A.P. (2009). Optimising ultrasound-mediated gene transfer (sonoporation) in vitro and prolonged expression of a transgene in vivo: potential applications for gene therapy of cancer. Cancer Lett..

[311] Sirsi S.R., Borden M.A. (2012). Advances in ultrasound mediated gene therapy using microbubble contrast agents. Theranostics.

[312] Sitta J., Howard C.M. (2021). Applications of ultrasound-mediated drug delivery and gene therapy. Int. J. Mol. Sci..

[313] Krut Z., Gazit D., Gazit Z., Pelled G. (2022). Applications of ultrasound-mediated gene delivery in regenerative medicine. Bioengineering (Basel).

[314] Clogston J.D., Foss W., Harris D., Oberoi H., Pan J., Pu E., Guzmán E.A.T., Walter K., Brown S., Soo P.L. (2024). Current state of nanomedicine drug products: an industry perspective. J. Pharm. Sci..

[315] Rathore A.S., Stevenson J.G., Chhabra H. (2021). Considerations related to comparative clinical studies for biosimilars. Expert Opin. Drug Saf..

[316] Gherghescu I., Delgado-Charro M.B. (2020). The biosimilar landscape: an overview of regulatory approvals by the EMA and FDA. Pharmaceutics.

[317] Kang S.L., Woo J.H., Kim N.H., Kwon J.Y., Kim S.M. (2023). Necessity of strengthening the current clinical regulatory for companion diagnostics: an institutional comparison of the FDA, EMA, and MFDS. Mol. Ther. Methods Clin. Dev..

[318] Lau F., Seifert R. (2025). Comparison of drug approvals of the FDA and EMA between 2013 and 2023. Naunyn Schmiedebergs Arch. Pharmacol..

